# Decoding Cretan Wines: Phenolic Profiling of Greek Indigenous Wine Varieties Using LC-QTOF-MS

**DOI:** 10.3390/molecules31050815

**Published:** 2026-02-28

**Authors:** Pelagia Lekka, Maria Dimitropoulou, Athanasia Rousali, Ana-Maria Kiose, Marianthi Basalekou, Nikolaos Thomaidis, Marilena Dasenaki

**Affiliations:** 1 Food Chemistry Laboratory, Department of Chemistry, National and Kapodistrian University of Athens, 15771 Athens, Greece; lekkap@chem.uoa.gr (P.L.); mardimi@chem.uoa.gr (M.D.); rousaliathanasia2001@gmail.com (A.R.); annemary.kiose@gmail.com (A.-M.K.); 2 Laboratory of Industrial Chemistry-Wine and Alcoholic Beverages, Department of Chemistry, National and Kapodistrian University of Athens, 15771 Athens, Greece; mbasalekou@chem.uoa.gr; 3 Analytical Chemistry Laboratory, Department of Chemistry, National and Kapodistrian University of Athens, 15771 Athens, Greece; ntho@chem.uoa.gr

**Keywords:** phenolic compounds, target screening, wine quality, statistical analysis, chemical profile

## Abstract

Crete’s rich heritage of indigenous wine grapes remains underexplored in terms of chemical composition, with many cultivars yet to be fully characterized. This study presents a comprehensive analysis of the phenolic profile of 67 monovarietal Cretan wines produced by 10 wineries (42 white, 25 red) from 12 varieties—eight white (Assyrtiko, Dafni, Malvazia, Melissaki, Moschato Spinas, Plito, Vidiano, and Vilana) and four red (Kotsifali, Liatiko, Mandilaria, and Romeiko). A targeted LC–QTOF–MS workflow covering 45 phenolic compounds (flavonoids and non-flavonoids) was applied. Varietal differences were assessed using heteroscedasticity-robust univariate statistics (Welch’s ANOVA with Games–Howell post hoc comparisons and effect-size estimation) and explored by multivariate analyses (PCA and HCA); cross-validated PLS-DA was used for descriptive classification, and MFA integrated the targeted phenolic matrix with classical indices (e.g., total phenolics, tannins, and color metrics). Red wines exhibited stronger variety-linked phenolic structuring than white wines, whereas white-wine differentiation was driven by a limited subset of marker phenolics. Given the central role of phenolic composition in overall wine quality, this study provides the first detailed phenolic characterization of 12 key indigenous Cretan grape varieties.

## 1. Introduction

Wine is a complex hydroalcoholic beverage composed of ethanol, water, sugars, organic acids, minerals, and numerous minor constituents such as volatile and phenolic compounds, which together determine its sensory and technological properties [1,2]. Among these, phenolic compounds are considered key determinants of wine quality, particularly in red wines, as they largely govern color, contribute to bitterness and astringency, and affect oxidative stability and aging behavior [2,3].

Wine phenolics form a structurally diverse family broadly grouped into flavonoids, including anthocyanidins, flavonols, flavan-3-ols, flavanones, flavones, and tannins, and non-flavonoids such as hydroxycinnamic and hydroxybenzoic acids, stilbenes, and simple phenols [1,4,5]. These classes of phenolics contribute to various wine attributes. Anthocyanins and tannins are primarily responsible for color and its stability, while tannins and flavan-3-ols influence astringency and bitterness [1,2,4]. Typical total polyphenol concentrations range from about 1–5 g/L in red wines and 0.2–0.5 g/L in white wines, consistent with the much higher extraction from skins and seeds during red wine fermentation and the more limited solid–liquid contact in white winemaking [1,4]. At the berry level, phenolic compounds are formed through the phenylpropanoid pathway, and the amounts that ultimately accumulate are strongly conditioned by genetic factors (variety, clone, rootstock), by the characteristics of the site (soil, topography, local climate), and by viticultural practices such as training system, canopy and yield management, irrigation, fertilization, and crop protection. During winemaking, their extraction from skins, seeds, and, when present, stems, together with subsequent oxidation, condensation, and polymerization reactions, further modifies their composition, so that the phenolic profile of the finished wine differs markedly from that of the original grapes [1,5,6].

Within the global wine sector, Greece is considered a small-to-medium producer: in 2024 its vineyard surface area totaled 92,833 ha, ranking 20th worldwide [7,8]. A distinctive feature of the Greek vineyard is its ampelographic diversity, with more than 400 indigenous grape varieties cultivated alongside international cultivars across nine main wine-growing regions on the mainland and islands [9,10].

Among these regions, Crete, the largest Greek island situated south of mainland Greece in the southern Aegean Sea, represents a principal island viticultural district, characterized by a long-standing winemaking heritage and a rich base of autochthonous cultivars [10,11]. Grape cultivation and wine production on the island date back to the Minoan period, and today, the island is home to several Protected Designation of Origin (PDO) and Protected Geographical Indication (PGI) zones, including PDO Peza, Archanes, Dafnes, and Sitia and PGI Crete, Heraklion, Rethymno, Chania, and Lasithi as recognized by the Hellenic Ministry of Rural Development and Food (2010) and the current European Commission registers [12]. These appellations are summarized in Supplementary Material Table S1, which details the Cretan PDO and PGI regions together with their classification, registration code, and date of recognition [13]. Structurally, the contemporary Cretan vineyard covers about 4200 ha, yields roughly 300,000 hL of wine per year, and comprises approximately 30–35 wineries, with Crete ranking as the 4th largest wine-growing region in Greece in terms of vineyard area [14,15,16].

In Crete, the Hellenic Ministry of Rural Development and Food, through Ministerial Decision 247771/04-03-2010, designates a broad set of indigenous and international grape varieties as recommended, permitted, or temporarily permitted for cultivation and wine production, including emblematic Cretan whites such as Vilana, Vidiano, Dafni, Thrapsathiri, and Plito and reds such as Liatiko, Kotsifali, Mandilaria, Thrapsa, and Romeiko, together with selected international cultivars (e.g., Chardonnay, Sauvignon Blanc, Syrah, and Cabernet Sauvignon) [12]. The full list of varieties by category is summarized in Supplementary Material Table S2. Recent ampelographic and SSR-based analyses have shown that traditional Cretan wine grapes such as Vidiano, Vilana, Thrapsathiri, Dafni, Liatiko, Kotsifali, Mandilaria, and Romeiko form a genetically diverse and partly unique gene pool, with considerable intra-varietal variation and numerous synonym–homonym cases, underscoring the richness and complexity of the island’s autochthonous germplasm [10,11].

Phenolic profiling is widely used to investigate wine quality attributes and to differentiate wines according to variety or origin, and several studies have applied this approach to Greek wines [5,17,18,19,20,21,22,23,24,25,26,27]. However, comprehensive phenolic datasets for Cretan indigenous varieties remain limited, with studies so far focusing mainly on Assyrtiko [17,21,28,29,30,31], Kotsifali [31,32], Liatiko [17,31,32,33], and Mandilaria [17,18,22,30,31] while comparable evidence is scarce for several other Cretan varieties such as Dafni, Malvazia, Melissaki, Moschato Spinas, Plito, Romeiko, Vidiano, and Vilana.

To address this gap, we provide a standardized targeted LC–QTOF–MS workflow to generate a varietal-level assessment of phenolic composition in monovarietal Cretan wines from 12 indigenous varieties spanning multiple vintages (mostly 2022–2023). Our study includes phenolic profiling of Assyrtiko, Dafni, Malvazia, Melissaki, Moschato Spinas, Plito, Vidiano, Vilana, and Romeiko (white wines) and Kotsifali, Liatiko, Mandilaria, and Romeiko (red wines). Following the detection and quantification of a comprehensive set of flavonoid and non-flavonoid phenolics, we (i) compare phenolic composition between Cretan red and white wines, (ii) evaluate varietal differentiation within each color group, and (iii) correlate the targeted phenolic fingerprint to routine compositional indices linked to phenolic expression (e.g., total phenolic content, color parameters, and tannin-related indices). Collectively, these data establish a reference framework for the phenolic landscape of Cretan monovarietal wines and support interpretation of how indigenous varieties express phenolic potential within the Cretan terroir.

## 2. Results and Discussion

### 2.1. Classical Oenological Parameters of Cretan Red and White Wines

Classical oenological parameters of the investigated Cretan red and white wines, measured by routine analytical methods, are summarized by variety in Tables S3 and S4 (Supplementary Material), respectively.

For red wines, Welch’s one-way ANOVA revealed statistically significant varietal effects for key phenolic-related indices, including PCI, tannins, and color attributes (color intensity and color hue), with large effect sizes despite unequal sample sizes. Mandilaria consistently exhibited the highest PCI, tannin concentration, and color intensity among the red varieties, in agreement with its reputation as a deeply colored, phenolic-rich cultivar [34,35]. However, the red-wine dataset encompasses a wider vintage range for Kotsifali, Liatiko, and Romeiko (including older vintages), whereas Mandilaria was represented predominantly by recent vintages; therefore, varietal comparisons for phenolic- and color-related indices should be interpreted with awareness of potential vintage and bottle-age contributions [36,37]. In contrast, routine classical analysis not directly linked to phenolic composition (e.g., SO_2_ fractions, pH, titratable acidity, and reducing sugars) showed limited varietal discrimination (η^2^ < 0.20).

For white wines, varietal differences were generally less pronounced, consistent with the lower phenolic load typical of white winemaking. Welch’s ANOVA highlighted significant differences in absorbance at 420 nm (A420), indicating varietal variation in browning-related optical properties. Folin–Ciocalteu TPC showed a moderate-to-large effect size (η^2^ = 0.684) but marginal significance (*p* = 0.068), likely influenced by limited sample sizes, while acidity, pH, and reducing sugars exhibited only small-to-moderate effects.

These findings provide a complementary technological context for the comprehensive phenolic profiling presented in the following sections.

### 2.2. Phenolic Composition of Cretan Red Wines

Across the Cretan red wines, targeted LC-QTOF-MS analysis quantified 35 non-anthocyanin phenolic compounds, grouped into 13 flavonoids and 22 non-flavonoids (Table 1). Nine compounds were not detected in any of the wine samples (hesperetin, apigenin, diosmetin, galangin, rutin, rosmarinic acid, chlorogenic acid, ferulic acid, and sinapic acid). Concentrations spanned almost three orders of magnitude, from low µg·L^−1^ levels for minor flavones and flavanones (which were frequently below the limit of quantification, <LOQ) to several tens of mg·L^−1^ for major constituents such as tyrosol (14.61–33.64 mg·L^−1^) and catechin (8.56–19.41 mg·L^−1^). Table 1 summarizes variety-wise mean ± SD for Kotsifali (*n* = 6), Liatiko (*n* = 12), Mandilaria (*n* = 4), and Romeiko (*n* = 3), together with Welch’s ANOVA results; given the unequal sample sizes and occasional high dispersion for some compounds, varietal comparisons should be interpreted cautiously.

From an oenological perspective, the quantified phenolic classes are directly linked to wine sensory and technological properties. In red wines, flavan-3-ols and their polymeric forms (tannins/proanthocyanidins) are major contributors to bitterness and astringency, whereas phenolic acids and other oxidizable phenols participate in oxidation reactions that influence color evolution and aging behavior [1,3,5].

Within the flavonoid fraction, flavan-3-ols were quantitatively dominant and showed clear varietal differences. Catechin ranged from 8.56 to 19.41 mg·L^−1^ (Welch *p* = 0.003; η^2^ = 0.337), with the highest mean in Mandilaria (19.410 ± 5.303 mg·L^−1^), followed by Kotsifali (15.005 ± 2.976 mg·L^−1^) and Liatiko (12.388 ± 5.580 mg·L^−1^), while Romeiko showed the lowest mean (8.563 ± 1.017 mg·L^−1^). Epicatechin ranged from 3.66 to 8.11 mg·L^−1^ (Welch *p* < 0.001; η^2^ = 0.236), with the highest mean in Liatiko, followed by Kotsifali and Mandilaria, which were very similar. Given that flavan-3-ols are precursors of condensed tannins and are central to mouthfeel, the higher catechin/epicatechin levels observed in Mandilaria and Liatiko are consistent with a greater potential contribution to bitterness and astringency compared with lower-flavan-3-ol profiles [1,3]. These flavan-3-ol levels lie at the lower end of the ranges reported for emblematic Greek wines such as Agiorgitiko wines from Nemea (catechin 18–38 mg·L^−1^, epicatechin 8–26 mg·L^−1^) and are clearly below those observed in Xinomavro wines from Naoussa (catechin 44–83 mg·L^−1^, epicatechin 18–59 mg·L^−1^) [38]. By contrast, they are comparable to or slightly higher than the catechin and epicatechin levels reported for Aidani Mavro and Mandilaria wines from Paros Island (Cyclades, Greece), where values around 6–8 mg·L^−1^ have been found [22]. Inter-study differences should be interpreted considering cultivar physiology as well as vinification practices that modulate phenolic extraction (e.g., maceration regime) and phenolic evolution during aging.

Other flavonoids occurred mainly at lower concentrations. Naringenin ranged from 9.82 to 15.01 mg·L^−1^ with the highest mean in Kotsifali and the lowest mean in Mandilaria, while pinobanksin ranged from 11.081 to 14.842 mg·L^−1^ and showed a similar magnitude across varieties. Flavonols and flavones were generally minor, with most compounds remaining below 1 mg·L^−1^. Quercetin was the most abundant flavonol (0.721–3.130 mg·L^−1^) and showed a marked decrease from Kotsifali (3.130 ± 0.694 mg·L^−1^) to Romeiko (0.721 ± 1.158 mg·L^−1^), although this effect did not reach conventional significance (Welch *p* = 0.068). Even at low concentrations, flavonols are technologically relevant because they can participate in co-pigmentation phenomena and thereby modulate color expression and stability in red wines [5]. Overall, the dominance of flavan-3-ols and the comparatively lower levels of flavonols and flavones are consistent with earlier HPLC-based characterizations of Greek red cultivars, including Thrapsa, Mandilaria, Xinomavro, and Agiorgitiko [18,22,39].

The non-flavonoid fraction was dominated by hydroxybenzoic acids, hydroxycinnamic acids and their derivatives, together with phenylethanoids and minor stilbenes and simple phenols. Gallic acid was the most abundant hydroxybenzoic acid (2.11–9.90 mg·L^−1^; Welch *p* = 0.01; η^2^ = 0.168). Similarly, syringic acid displayed significant varietal differences (Welch *p* = 0.004), being <LOQ in Kotsifali but quantifiable in the other varieties. Other hydroxybenzoic acids were present mostly at sub-mg·L^−1^ levels like syringic acid, 3/4-hydroxybenzoic acid, and protocatechuic acid. Protocatechuic acid ranged from 0.223 to 0.430 mg·L^−1^ and showed a significant varietal effect (Welch *p* = 0.037; η^2^ = 0.094). Overall, these concentrations lie toward the lower end of the broad ranges reported for Greek red wines from cultivars such as Agiorgitiko and Xinomavro and are consistent with earlier surveys of Greek red varieties [18,19,22].

Among hydroxycinnamates, caffeic acid ranged from 2.50 to 5.48 mg·L^−1^ and differed significantly among varieties, with the highest mean in Liatiko 5.484 ± 2.635 mg·L^−1^ and similarly elevated values in Romeiko 5.087 ± 2.127 mg·L^−1^. p-Coumaric acid reached its highest mean in Romeiko 1.323 ± 0.918 mg·L^−1^ and Liatiko 1.009 ± 0.986 mg·L^−1^ although the varietal effect was not significant. Esterified phenolics were prominent and variable: ethyl gallate (3.13–7.76 mg·L^−1^) and ethyl caffeate (2.70–35.45 mg·L^−1^), with the highest mean in Romeiko, exhibited very high dispersion. This pattern is consistent with the known sensitivity of phenolic esters to winemaking and aging conditions and the high between-sample variability often observed for these compounds in red wines [19]. Hydroxycinnamates and related oxidizable phenols are also key substrates in oxidative pathways, linking their variability to differences in oxidation-driven evolution during storage and aging [1,3,5].

Phenylethanoids were quantitatively important across all varieties. Tyrosol (14.61–33.64 mg·L^−1^) with the highest mean in Mandilaria, differed significantly among varieties. Hydroxytyrosol reaching its highest mean in Romeiko (1.56–2.50 mg·L^−1^) occurred at levels comparable to those described for Mediterranean red wines (tyrosol 12–74 mg·L^−1^, hydroxytyrosol 0.72–3.9 mg·L^−1^). Both are secondary metabolites associated with yeast-driven alcoholic fermentation and have attracted interest for their bioactive potential [22,40,41]. Stilbenes and simple phenols were, as expected, present at low concentrations: resveratrol occurred at low levels (0.233–0.516 mg·L^−1^; Welch *p* = 0.097), with higher means in Liatiko 0.516 ± 0.453 mg·L^−1^ and Kotsifali 0.450 ± 0.285 mg·L^−1^. Given its predominant localization in grape skins, resveratrol levels are influenced by the extent of skin–must contact and may vary with cultivar, growing conditions, and vinification practices [41]. Catechol remained predominantly below the limit of quantification (<LOQ) across most varieties, with only minor trace detections (e.g., 0.102 mg·L^−1^ in Mandilaria) consistent with the generally minor contribution of these compounds to the non-anthocyanin phenolic pool in red wines [41].

#### 2.2.1. Univariate Varietal Differences in Phenolic Composition

Univariate varietal effects were evaluated using Welch’s one-way ANOVA (variety as a fixed factor) with Games–Howell post-hoc comparisons, appropriate for unequal group sizes and heteroscedasticity. Effect sizes are reported as eta-squared (η^2^) (Table 1).

**Figure 1:**
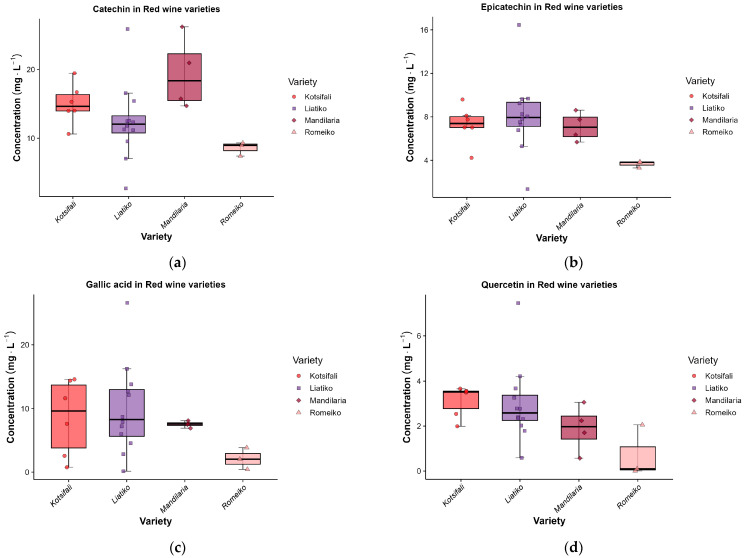
Box plots of (**a**) Catechin (**b**) Epicatechin (**c**) Gallic acid and (**d**) Quercetin in Cretan red wine varieties.

Across the quantified phenolics, nine compounds showed significant varietal differences (Welch *p* < 0.05; Table 1), suggesting that varietal discrimination is mainly driven by a limited subset of compounds rather than uniform shifts across the entire phenolic profile. The strongest separation was observed for flavan-3-ols: catechin (*p* = 0.003; η^2^ = 0.337) and epicatechin (*p* < 0.001; η^2^ = 0.236) concentrations are significantly lower in the Romeiko variety, with catechin distinguishing Romeiko from Kotsifali and epicatechin distinguishing Romeiko from all other varieties. Although Mandilaria showed higher median catechin values in the boxplot, post-hoc testing did not support consistent separation from the other varieties (Mandilaria and Liatiko displayed intermediate levels), likely reflecting limited sample size and within-variety dispersion. These patterns are reflected in the boxplots (Figure 1), where Romeiko exhibits both low central tendency and limited dispersion, while Liatiko displays wider variability.

Within flavonols, kaempferol differed significantly among cultivars (*p* = 0.031; η^2^ = 0.357), driven mainly by higher concentrations in Liatiko compared to Mandilaria (b vs. a), with Kotsifali and Romeiko occupying intermediate groups (ab). Among non-flavonoids, gallic acid showed a moderate varietal effect (*p* = 0.010; η^2^ = 0.168), primarily reflecting lower levels in Romeiko compared with Liatiko (b vs. a). Liatiko also presented higher concentrations of caffeic acid than Kotsifali (*p* = 0.045; η^2^ = 0.312), while Mandilaria and Romeiko presented intermediate concentrations. Three additional markers contributed to varietal separation within the benzoate/acid pool: protocatechuic acid (*p* = 0.037; η^2^ = 0.094) exhibited a statistically detectable but small effect with no resolved pairwise separation in the post-hoc lettering (all “a”). Syringic acid emerged as a strong discriminator (*p* = 0.004; η^2^ = 0.666), with Kotsifali remaining below the limit of quantification (<LOQ) while Liatiko and Mandilaria formed an upper group (“a”) relative to Romeiko (“b”). Furthermore, protocatechuic acid ethyl ester (*p* = 0.001) clearly differentiated Mandilaria (lower) from Liatiko (higher) (a vs. b). Finally, tyrosol exhibited one of the largest varietal effects (*p* = 0.015; η^2^ = 0.457), with Mandilaria forming the upper group compared to Kotsifali and Liatiko (a vs. b), supporting a phenylethanoid-enriched signature for Mandilaria.

Within the red cultivars Liatiko, Kotsifali, Mandilaria, and Romeiko, typicity aligned mainly with a flavanol and phenylethanoid-rich pattern. Liatiko (best represented) combined the highest epicatechin (8.110 ± 3.469 mg·L^−1^) with the numerically highest resveratrol (0.516 ± 0.453 mg·L^−1^), alongside prominent gallic and caffeic acids and flavonols such as kaempferol and quercetin. Kotsifali closely tracked the major flavanols (catechin 15.005 ± 2.976 mg·L^−1^; epicatechin 7.286 ± 1.772 mg·L^−1^) while showing strong naringenin and pinobanksin together with higher protocatechuic acid ethyl ester (0.463 ± 0.293 mg·L^−1^). Despite smaller subsets, Mandilaria concentrated the highest mean catechin (19.410 ± 5.303 mg·L^−1^) and tyrosol (33.638 ± 5.967 mg·L^−1^), whereas Romeiko was distinguished by a hydroxycinnamate/phenylethanoid signature led by ethyl caffeate (35.452 ± 55.328 mg·L^−1^), with accompanying p-coumaric acid and hydroxytyrosol, although this inference should be interpreted with caution given the limited Romeiko subset (*n* = 3).

#### 2.2.2. Heatmap-Based Clustering and Multivariate Separation

Prior to multivariate analysis, low-frequency variables were filtered out. Specifically, phenolic compounds not detected in any sample or detected in fewer than four samples were excluded, resulting in a curated dataset of 23 phenolic compounds used for subsequent chemometric analyses.

**Figure 2:**
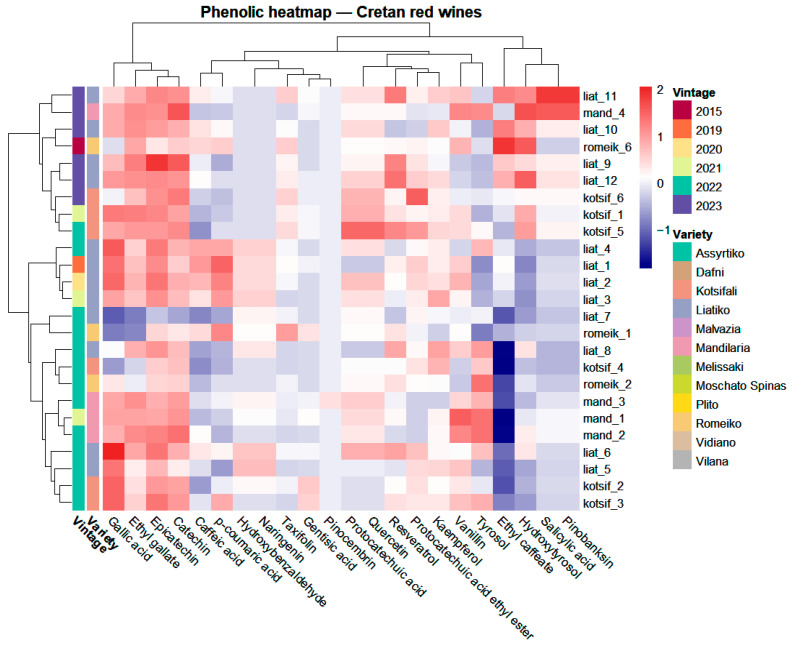
Hierarchical clustering heatmap of phenolic composition in Cretan red wine varieties.

The resulting phenolic matrix (log-transformed and Pareto-scaled) was explored by hierarchical clustering analysis (HCA) using Euclidean distance and Ward’s linkage, with simultaneous clustering of samples and variables and visualization as a heatmap annotated by variety, region, vintage, and winery (Figure 2).

The heatmap revealed a dominant co-varying block comprising ethyl gallate (benzoic acid derivative) and gallic acid (benzoic acid), together with catechin and epicatechin (flavan-3-ols) and quercetin (flavonol). This block displayed predominantly positive Z-scores across most samples, indicating a shared “core” phenolic pattern among the Cretan red wines. Two samples (one Romeiko and one Liatiko) showed a concerted reduction across the entire block and emerged as clear outliers. Within the Kotsifali variety, sample kotsif_4 further deviated by exhibiting selectively low Z-scores for ethyl gallate and gallic acid, despite the generally elevated behavior of the block. The remaining variables formed a broader assemblage with structured sub-patterns: hydroxycinnamate-related features (ethyl caffeate, caffeic acid, and p-coumaric acid) were generally low with sporadic increases in a few wines; vanillin (benzoic aldehyde) and tyrosol (phenylethanol derivative) were comparatively enriched in Mandilaria; and a set including salicylic acid (benzoic acid), hydroxytyrosol and resveratrol (phenylethanol derivative and stilbene), naringenin (flavanone), and pinobanksin/taxifolin (flavanonol) showed higher levels in subsets of Kotsifali and Liatiko. In contrast, eriodictyol was consistently below the limit of quantification (<LOQ) across all white varieties, while pinocembrin and gentisic acid remained <LOQ in nearly all wines, with pinocembrin quantifiable only in the Plito variety. Furthermore, markers such as hydroxybenzaldehyde (benzoic aldehyde), kaempferol (flavonol), protocatechuic acid (benzoic acid), and protocatechuic acid ethyl ester (benzoic acid derivative were only sporadically or at trace levels, while protocatechuic acid exhibited a consistent but low-level presence (ranging from <LOQ to 0.219 mg·L^−1^) across the analyzed Cretan white wines.

At the sample level, the dendrogram indicated a primarily variety-driven structure (Figure 2). Mandilaria wines formed the most compact branch, consistent with their comparatively uniform phenolic fingerprint and their enrichment in the vanillin/tyrosol pattern. Liatiko showed the greatest dispersion and partial overlap with Romeiko and Kotsifali, suggesting higher within-variety heterogeneity and/or stronger modulation by sample-specific factors. Vintage annotations were interspersed within the major branches rather than forming vintage-specific groupings, supporting a weaker vintage imprint relative to variety for the phenolic space captured here.

**Figure 3:**
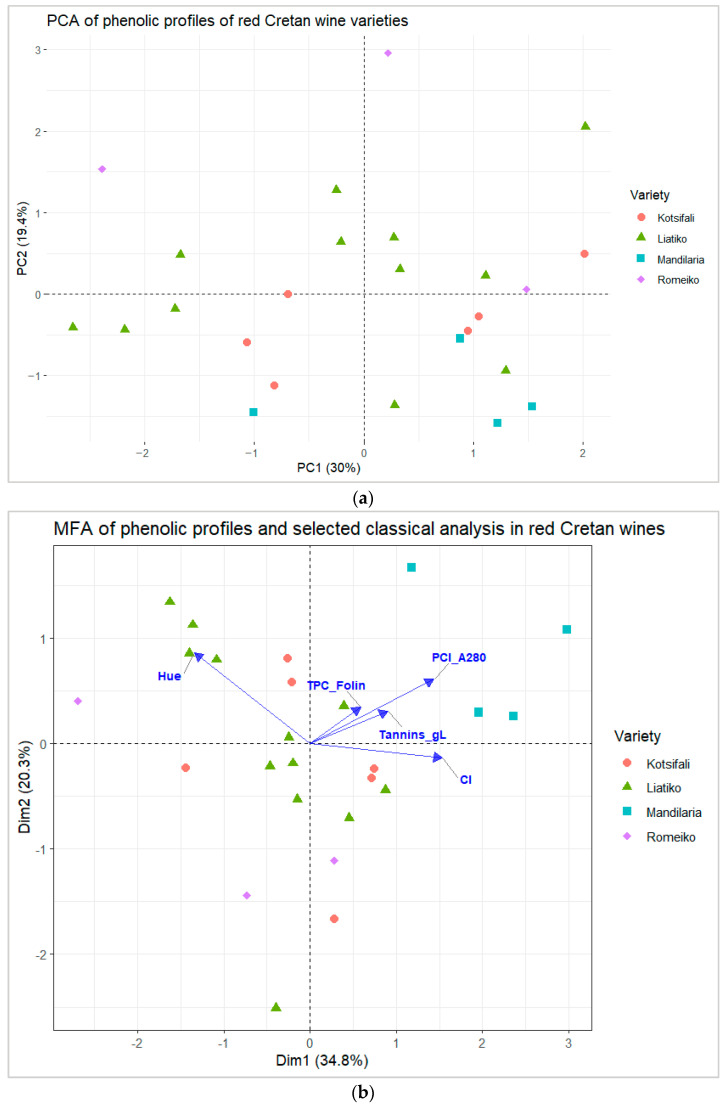
Multivariate analysis of Cretan red wines by variety: (**a**) Principal component analysis (PCA) score plot based on phenolic composition, with 95% confidence ellipses for each variety (Kotsifali, Liatiko, Mandilaria, and Romeiko); (**b**) Multiple factor analysis (MFA) score plot integrating phenolic composition with selected classical parameters (phenolic content index at 280 nm, tannins, and total phenolic content by Folin–Ciocalteu), with variety-specific confidence ellipses and loading vectors for the classical variables.

These trends were corroborated by PCA performed on the same preprocessed matrix (Figure 3) and diagnostic and cross-validation tests, which are detailed in Supplementary Material (Supplementary Method S1, Tables S5 and S6). The first two components accounted for 49.4% of the total variance (PC1 = 30.0%, PC2 = 19.4%) and reproduced the varietal structure observed by HCA: Mandilaria clustered tightly, with the exception of sample ‘mand_4’ from the 2023 vintage, which exhibited more variability and slightly separated along PC2 (negative scores). The other Mandilaria samples from 2021 and 2022 clustered closely together. Kotsifali was centered closer to the origin with moderate dispersion, whereas Liatiko exhibited broad spread and overlap with Romeiko, which also showed wide dispersion and included extreme observations. The single Romeiko 2015 sample did not exhibit a consistent decrease in quantified phenolics compared to Romeiko 2022 samples, and Liatiko wines across 2019–2023 likewise showed non-monotonic vintage-related variation, suggesting modulation rather than systematic degradation. Overall, PCA and HCA were concordant, indicating that multivariate separation is driven by coordinated shifts across phenolic subclasses/blocks rather than isolated single-compound effects, with variety emerging as the dominant source of structure in this dataset.

To complement the unsupervised analyses, a supervised PLS–DA model (Supplementary Figure S1a) was fitted to the same log-transformed, Pareto-scaled phenolic matrix and evaluated by cross-validation. The cross-validated BER decreased with the number of components (0.6308, 0.5792, and 0.4400 for 1–3 components), indicating improved though still moderate discrimination across varieties (Supplementary Table S7). In the corresponding score plot, Mandilaria showed the most compact separation, while Kotsifali and Liatiko displayed partial overlap. Full PLS–DA outputs, including the descriptive (in-sample) confusion matrix and VIP-ranked predictors, are provided in the Supplementary Material (Supplementary Tables S8 and S9).

#### 2.2.3. Multiple Factor Analysis (MFA): Linking Phenolic Composition with Classical Indices

To evaluate whether the variety-driven structure observed in the targeted phenolic dataset is reflected in routine oenological analysis of overall “phenolic richness” and optical properties, Multiple Factor Analysis (MFA) was performed. This analysis combined two quantitative blocks measured on the same wines: (i) the log-transformed, Pareto-scaled targeted phenolic data matrix and (ii) five classical indices, namely, phenolic content index at 280 nm (PCI, A_280_), tannins (g/L), total phenolic content by Folin–Ciocalteu (mg·L^−1^ GAE), color intensity (I), and color hue (T) (Figure 3b). PCI (A280) was used as a rapid spectrophotometric proxy for UV-absorbing phenolic constituents and provides a rapid global estimate of wine phenolics that share aromatic chromophores absorbing near 280 nm [42]. The tannin assay quantifies the polymeric phenolic fraction, primarily condensed tannins and proanthocyanidins, which contribute to structure and astringency in red wines. While methods for quantification vary, tannin levels are best interpreted comparatively within-study [43,44]. Folin–Ciocalteu TPC was retained as a widely reported global benchmark, while interpreted cautiously due to its broader responsiveness to reducing compounds beyond phenolics [42,43,45,46].

Color metrics were incorporated because wine appearance is a key integrative outcome of phenolic extraction and pigment chemistry and can provide complementary information to bulk phenolic indices [47,48]. In red wines, color intensity is closely linked to the abundance and extractability of colored phenolics, particularly anthocyanins, and to pigment stabilization phenomena involving other phenolics (e.g., co-pigmentation and polymeric pigment formation) [48,49]. In the present dataset, color intensity differed at the threshold of significance among varieties (Welch *p* = 0.0500; η^2^ = 0.616; range 3.4–12.3) and followed the same overall trend as PCI and tannins, with the highest mean value observed for Mandilaria (12.3 ± 2.5) (Table S6).

On the other hand, hue (T), which measures the balance between red and yellow tones, is influenced by various factors, including grape variety, winemaking processes, and aging. While hue can indicate aging-related chemical transformations, such as the shift from red to yellow as anthocyanins polymerize, it is also influenced by other factors like pH and copigmentation. In this study, Mandilaria had the lowest hue (T = 0.79 ± 0.07), reflecting more red tones, while Romeiko, with the highest hue (T = 1.37 ± 0.52), displayed more yellow tones, suggesting that hue is influenced not only by aging but also by the specific phenolic composition of grapes and winemaking techniques. Additionally, pH values were measured (ranging from 3.42 to 3.54 across the four varieties), which can influence both color intensity and hue. For example, wines with a lower pH tend to retain more intense red hues, as the flavylium form of anthocyanins is more stable under acidic conditions [50].

MFA is well suited for integrating such heterogeneous but related datasets because it balances the contribution of each block via block-wise normalization before computing the compromise space [51,52]. In the present data, MFA reproduced the varietal organization previously observed in the phenolic PCA and provided an interpretable linkage to classical indices (Figure 3b). The first MFA dimension (Dim1, 34.8%) reflected a dominant “phenolic richness and extraction” gradient, as shown by the strong co-orientation of PCI (A_280_) and tannins toward positive Dim1, together with color intensity and, to a lesser extent, Folin–Ciocalteu TPC. Consistent with this configuration, Mandilaria projected clearly to the positive side of Dim1 and exhibited the highest mean PCI (84.7 ± 19.7; Welch *p* = 0.0050; η^2^ = 0.705) and tannins (7.36 ± 1.46 g/L; Welch *p* < 0.001; η^2^ = 0.755), along with the highest color intensity (12.3 ± 2.5) (Table S6). At the opposite side of Dim1, Romeiko aligned with lower global indices (PCI 33.7 ± 2.1; tannins 1.83 ± 0.15 g/L) and showed the lowest mean color intensity (3.4 ± 2.7). Notably, the two most represented varieties, Kotsifali (*n* = 6) and Liatiko (*n* = 12), occupied intermediate positions near the compromise-space origin with partial overlap (Figure 3b), reflecting their moderate mean values for PCI (42.0 ± 9.9 and 42.2 ± 9.6) and tannins (3.10 ± 1.30 and 3.05 ± 0.81 g/L) relative to Mandilaria and Romeiko. Their dispersion around the center, visible in both dimensions, indicates meaningful intra-varietal heterogeneity, particularly for the color metrics (I and T), where Kotsifali and Liatiko share similar mean hue (1.10 ± 0.13 and 1.15 ± 0.12) and moderate color intensity (6.6 ± 2.6 and 5.4 ± 2.0), but span a broader region of the MFA space than Mandilaria (Figure 3b). This spread is consistent with the expectation that, for cultivars with larger representation, differences in vineyard site, vintage, and vinification practices can modulate extraction and pigment stabilization pathways, generating within-variety variability that is not fully captured by a single bulk index [53,54].

Although Folin–Ciocalteu TPC did not reach significance at *p* = 0.05 (Welch *p* = 0.0980), it displayed a non-trivial effect size (η^2^ = 0.483) and followed the same directional trend (highest mean in Mandilaria: 4310 ± 1620 mg·L^−1^ GAE), supporting its use as a complementary, nonspecific descriptor within the integrated space [50]. The second dimension (Dim2, 20.3%) captured a pattern driven primarily by hue (vector-oriented opposite to the phenolic-richness cluster of PCI/tannins/CI), indicating that variation in tonal balance (red vs. yellow/brown) contributes additional structure beyond bulk “quantity” metrics. This is consistent with the multifactorial determinants of hue, which can vary independently of overall phenolic abundance due to differences in pigment profiles, copigmentation, and derived pigment formation [53,54]. In this context, Mandilaria combined high phenolic indices with low hue (T = 0.79 ± 0.07), whereas Romeiko displayed higher hue (T = 1.37 ± 0.52), suggesting varietal differentiation in pigment chemistry rather than a simple concentration effect.

Overall, MFA was concordant with the unsupervised multivariate patterns, indicating that the main separation among Cretan red varieties is driven by a phenolic richness/tannin gradient. Color-related, particularly hue, provided an additional, chemically meaningful dimension related to pigment composition and transformation chemistry. Mandilaria is characterized by consistently higher phenolic indices and deeper color intensity, Romeiko by lower phenolic indices and lighter/tonally shifted color properties, and Kotsifali and Liatiko occupy intermediate positions with greater internal variability that contributes substantially to the compositional diversity captured by the integrated model.

### 2.3. Phenolic Composition of Cretan White Wines

Targeted LC–QTOF–MS quantified 35 non-anthocyanin phenolic compounds in the Cretan white wines, comprising 14 flavonoids and 21 non-flavonoids (Table 2). In contrast to the red wines, several analytes were found at trace concentrations strictly below the limit of quantification (<LOQ) or were detected only sporadically (often in a single wine within a variety and/or absent from most varieties). This includes compounds such as eriodictyol, pinocembrin, hesperetin, sakuranetin, luteolin, galangin, chrysin, catechol, and ferulic acid, indicating a largely trace-level and wine-dependent distribution under white-wine vinification conditions. This pattern is consistent with the view that many flavones, flavonols, and several phenolic acids are intrinsically low in white wines and are highly sensitive to extraction conditions (pressing and skin contact) and oxidative losses during processing [55,56]. Importantly, white-wine phenolic profiles are often dominated by hydroxycinnamate derivatives (frequently present as tartaric esters), and cross-study compositional comparisons can be strongly influenced by whether these major forms are included in the targeted panel, as well as by sample handling and oxygen exposure prior to analysis [55,56]. Table 2 reports the mean ± SD concentrations of each compound across the nine Cretan white varieties (Assyrtiko, Dafni, Malvazia, Melissaki, Moschato Spinas, Plito, Romeiko, Vidiano, and Vilana). Given that some varieties were so rare that we could not obtain more than two samples, it is important to note that the small sample sizes (*n* = 2–4) and the occurrence of single detections for some compounds limit the statistical reliability of varietal inferences.

One of these varieties, Melissaki, is a rare indigenous white grape variety of Crete that has been maintained in limited plantings and has been considered at risk of disappearance. In the present dataset, the Melissaki wine was produced by skin–juice maceration (orange-wine-style vinification), a processing choice known to enhance the extraction of skin- and seed-derived phenolics into the must. Accordingly, Melissaki was treated as a process-distinct sample and was excluded from most between-variety comparisons among conventionally vinified white wines to avoid confounding varietal effects with maceration-driven extraction effects. Varietal concentration summaries and Welch’s ANOVA outputs for the white-wine dataset excluding Melissaki are provided in the Supplementary Material (Table S10).

Within the flavonoid fraction, flavan-3-ols were consistently present and constituted the most robust component of the flavonoid pool. Catechin and epicatechin were detected in all varieties, with varietal mean concentrations of 0.744–15.909 and 0.264–5.470 mg·L^−1^, respectively; both exhibited pronounced varietal dependence (catechin: Welch *p* < 0.001, η^2^ = 0.651; epicatechin: Welch *p* = 0.01, η^2^ = 0.659). Melissaki showed the highest varietal means for both flavan-3-ols (catechin 15.909 mg·L^−1^; epicatechin 5.470 mg·L^−1^), this elevation may be explained, at least in part, by the fact that Melissaki was the only white wine in the set produced using a red-wine-style vinification with skin-juice maceration (orange-wine-type processing), which promotes extraction of skin and seed-derived phenolics (including flavan-3-ols) into the fermenting must [57,58,59]. Such enrichment is consistent with the enhanced extraction of skin- and seed-derived flavan-3-ols during prolonged maceration in macerated (“orange”) white wines, where catechin and related phenolics are frequently among the dominant components [58,59]. Excluding Melissaki, catechin concentrations were highest in Assyrtiko (6.078 ± 4.340 mg·L^−1^), followed by Vidiano (4.005 ± 1.993 mg·L^−1^) and Plito (3.494 ± 3.476 mg·L^−1^). For epicatechin, the highest concentrations were observed in Assyrtiko (1.675 ± 1.405 mg·L^−1^) and Vidiano (1.316 ± 0.718 mg·L^−1^), followed by Plito (1.145 ± 1.088 mg·L^−1^). According to Kallithraka et al. (2001), Greek white wines, including Assyrtiko, Moschofilero, Robola, Athiri, and Roditis, can show highly variable flavan-3-ol levels depending on cultivar and vinification, with catechin ranging from 0.0 to 100.0 mg·L^−1^ and epicatechin from 0.0 to 61.3 mg·L^−1^ [17]. A larger targeted LC–QTOF–MS survey by Tzachristas et al. (2021) reported catechin concentrations ranging from 0.71 to 16 mg·L^−1^ and epicatechin from below the detection limit (<LOD) to 16 mg·L^−1^ across 97 monovarietal Greek white wine samples, including Assyrtiko, Moschofilero, Malagousia, and Savatiano [29].

Flavonols and flavones were generally minor in the present Cretan set. Compounds such as kaempferol, luteolin, and chrysin were predominantly below the limit of quantification (<LOQ) or not detected in most varieties, with only sporadic trace-level detections (e.g., up to 0.343 mg·L^−1^ for kaempferol and 0.339 mg·L^−1^ for luteolin). Taxifolin was also present at low levels, ranging from <LOQ up to 0.243 ± 0.172 mg·L^−1^. Quercetin was generally low or <LOQ in most varieties; however, moderate levels were observed in Vidiano (0.158 ± 0.153 mg·L^−1^), indicating that flavonols are not uniformly negligible across all white wines. The flavanone/flavanonol fraction was quantitatively prominent. Naringenin concentrations ranged from 3.531 ± 2.839 mg·L^−1^ in Moschato Spinas to 34.093 ± 38.419 mg·L^−1^ in Plito, followed by Assyrtiko (21.058 ± 30.591 mg·L^−1^) and Vidiano (19.333 ± 18.231 mg·L^−1^). Pinobanksin concentrations followed a similar order of magnitude across these varieties [55,56].

The non-flavonoid fraction was dominated by phenolic acids and their derivatives, together with phenylethanoids and minor phenolic aldehydes and stilbenes. Among benzoic acids, gallic acid was the most abundant, ranging from 0.121 ± 0.088 mg·L^−1^ in Plito to 6.494 ± 6.681 mg·L^−1^ in Vilana, showing the highest concentration, while gentisic and protocatechuic acids occurred mainly at sub-mg·L^−1^ levels. Although most hydroxybenzoic acids occurred at sub-mg·L^−1^ levels, 3-hydroxybenzoic acid reached 0.507 mg·L^−1^ in Vilana (*n* = 1). Hydroxycinnamic acids were led by caffeic acid (ranged from 0.809 ± 0.385 mg·L^−1^ in Moschato Spinas to 9.374 ± 2.257 mg·L^−1^ in Vilana) and and p-coumaric acid, which was highest in Assyrtiko (1.345 ± 1.203 mg·L^−1^) and Vidiano (0.811 ± 0.830 mg·L^−1^), while ferulic acid was detected only occasionally. A distinctive feature of this dataset was the high and highly variable concentration of the hydroxycinnamate ester ethyl caffeate (3.018–69.967 mg·L^−1^), which differed significantly among varieties (Welch *p* = 0.013) and peaked in Plito (69.967 ± 12.451 mg·L^−1^). Hydroxycinnamates play a key role in white-wine oxidation chemistry, and elevated levels of ethyl caffeate likely reflect cultivar-dependent precursor availability and/or fermentation and aging conditions that promote ester formation or persistence, which may influence the antioxidant properties of the wine [55,56]. Fermentation-derived phenylethanoids were quantitatively important across all cultivars, with tyrosol reaching 17.792 ± 4.916 mg·L^−1^ in Malvazia and hydroxytyrosol reaching 2.822 ± 0.641 mg·L^−1^ in Plito (excluding Melissaki). Tyrosol and hydroxytyrosol are among the most consistently detected phenolics in white wines and are widely regarded as fermentation-related phenylethanoids, which helps explain their ubiquitous presence even under limited skin-contact conditions. Their levels in our Cretan set are broadly comparable to those previously reported for Greek white wines (tyrosol ~10–15 mg·L^−1^; hydroxytyrosol ~0.53–1.8 mg·L^−1^) [29]. Their occurrence is also relevant from a bioactivity perspective, as tyrosol and especially hydroxytyrosol are well-recognized antioxidant phenolics in foods and have been linked to additional biological effects (e.g., anti-inflammatory and cardiometabolic-related endpoints) in the broader dietary literature [41,60,61].

Finally, resveratrol was quantified at the highest levels in Dafni, reaching 0.322 ± 0.240 mg·L^−1^, Melissaki 0.162 ± 0.050 mg·L^−1^, Vilana 0.226 ± 0.146 mg·L^−1^ and Vidiano 0.104 ± 0.079 mg·L^−1^. In several other white varieties, resveratrol remained below the limit of quantification. These generally low concentrations across the dataset are consistent with generally reduced stilbene extraction in white vinifications due to limited skin contact [55,56]. Nevertheless, the quantifiable concentrations observed here fall within the broad range reported for white wines [62].

Overall, the targeted phenolic profiles revealed clear variety-linked patterns among Cretan white wines, while also underscoring the strong influence of vinification on extractability. Melissaki should be interpreted separately because its red-wine-style skin contact likely explains its markedly higher extraction-derived flavan-3-ols. Excluding Melissaki, Assyrtiko and Vidiano showed the most consistently phenolic-rich profiles among the conventionally vinified whites, with higher mean flavan-3-ols (and, for Assyrtiko, the highest p-coumaric acid), whereas Moschato Spinas tended toward lower flavonoid levels (including the lowest catechin and naringenin). Plito was distinctive for a hydroxycinnamate/fermentation-related signature, combining the highest ethyl caffeate, the highest naringenin, and the highest hydroxytyrosol, while Malvazia stood out for the highest tyrosol. Among the remaining varieties, Vilana showed the highest caffeic acid and relatively high ethyl caffeate, Dafni and Vilana reached the highest resveratrol, and Romeiko (white vinification) displayed intermediate phenolic levels overall but comparatively elevated resveratrol relative to several conventional whites.

#### 2.3.1. Univariate Varietal Differences in Phenolic Composition

Univariate varietal effects in white wines were evaluated using Welch’s one-way ANOVA (variety as a fixed factor) followed by Games–Howell post-hoc comparisons, which are appropriate for unequal group sizes and heteroscedasticity. Effect sizes are reported as eta-squared (η^2^) (Table 2).

**Figure 4:**
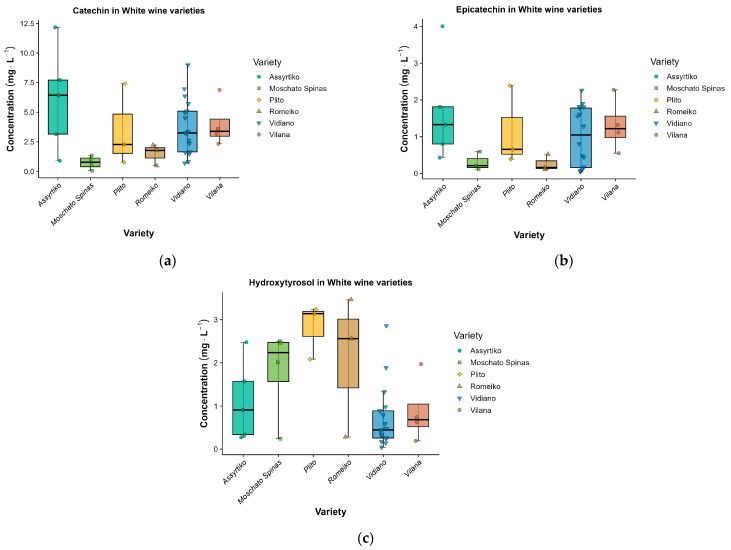
Box plots of (**a**) Catechin (**b**) Epicatechin (**c**) Hydroxytyrosol in Cretan white wine varieties.

Across the quantified phenolics, only three compounds showed significant varietal differences (Welch *p* < 0.05; Table 2), showing that only a very small number of phenolics account for most of the observed between-variety differences in the white wines. The strongest effects were observed for flavan-3-ols: catechin (*p* < 0.001; η^2^ = 0.651) and epicatechin (*p* = 0.010; η^2^ = 0.659). For catechin (Figure 4a), the post-hoc lettering indicates a clear low–high contrast driven by Moschato Spinas (lowest; “b”) relative to varieties in the upper group (e.g., Dafni and Vidiano; “a”), while several cultivars fall into intermediate overlapping groups (“ab”). Melissaki displayed the highest mean catechin and epicatechin levels, consistent with enhanced extraction of flavanols under increased skin/solid contact during vinification, whereas Moschato Spinas showed consistently low flavanol abundance. For epicatechin (Figure 4b), although the global Welch test was significant, pairwise separation was not resolved in the lettering (all “a”), suggesting that high within-variety dispersion and small n for several cultivars limited post-hoc discrimination despite an overall varietal signal.

Among non-flavonoids, only one esterified phenolic marker contributed to varietal separation. Ethyl caffeate (*p* = 0.013; η^2^ = 0.198) differentiated Plito (highest; “a”) from the lower group (Dafni and Vidiano; “b”), with the remaining varieties largely overlapping (“ab”), pointing to a cultivar-linked tendency in hydroxycinnamate-ester abundance.

Given that Melissaki was vinified under extended skin contact (orange-wine style), a sensitivity analysis was performed by repeating the univariate comparisons after excluding Melissaki (Table S8) to evaluate the robustness of varietal effects within the conventionally vinified white-wine set. Under this approach, the main signals were retained, with catechin, epicatechin, ethyl caffeate, and resveratrol remaining significant. Notably, hydroxytyrosol (Figure 4c) emerged as a key discriminator (*p* < 0.001; η^2^ = 0.462), driven by higher levels in Malvazia (2.257 ± 0.206; group “a”) relative to Dafni (0.296 ± 0.163; group “b”) and Vidiano (0.716 ± 0.729; group “b”), with the remaining varieties largely intermediate (“ab”). Protocatechuic acid ethyl ester remained statistically significant (*p* = 0.005) but with negligible effect size (η^2^ ≈ 0). Overall, exclusion of Melissaki reduced flavanol effect sizes but supports that the principal varietal contrasts are not solely driven by orange-wine vinification.

Vidiano, the most represented cultivar in the present study (*n* = 17), provided a stable reference profile for the major flavanols (catechin and epicatechin) and for the hydroxycinnamate and phenylethanoid space (ethyl caffeate 22.575 ± 31.160 mg·L^−1^; hydroxytyrosol 0.716 ± 0.729 mg·L^−1^), whereas Assyrtiko expressed the most flavanol-forward behavior among these whites (catechin 6.078 ± 4.340 mg·L^−1^; epicatechin 1.675 ± 1.405 mg·L^−1^). Vilana (*n* = 4) tended toward higher benzoate-ester expression (protocatechuic acid ethyl ester 0.170 ± 0.174 mg·L^−1^) and also showed a relatively strong stilbene signal (resveratrol 0.226 ± 0.146 mg·L^−1^). Plito emerged as chemically distinctive and was defined by the highest ethyl caffeate (69.967 ± 12.451 mg·L^−1^) together with elevated hydroxytyrosol, indicating that hydroxycinnamate-ester and phenylethanoid features are central to its fingerprint; Malvazia similarly expressed a pronounced phenylethanoid signal (notably hydroxytyrosol 2.257 ± 0.206 mg·L^−1^). Among the less represented subsets, Dafni (*n* = 2) was characterized by a comparatively high stilbene signal (resveratrol 0.322 ± 0.240 mg·L^−1^), whereas Romeiko (white vinification; *n* = 3) combined a high resveratrol signal (0.124 ± 0.010 mg·L^−1^) with elevated protocatechuic acid ethyl ester. Moschato Spinas also showed substantial ethyl-caffeate levels, reinforcing that, in these white wines, cultivar information is captured most strongly by a small set of flavanol, hydroxycinnamate-ester, phenylethanoid, and stilbene markers, interpreted with appropriate caution for the least represented subsets. Finally, Melissaki exhibited markedly elevated flavan-3-ols (catechin, epicatechin) together with higher flavonols (quercetin, kaempferol) and a strong phenylethanoid signature (hydroxytyrosol and tyrosol), resulting in a phenolic-forward profile that more closely approaches the concentration range typically associated with skin-influenced (orange-style) vinification than with standard white-wine processing.

#### 2.3.2. Heatmap-Based Clustering and Multivariate Separation

**Figure 5:**
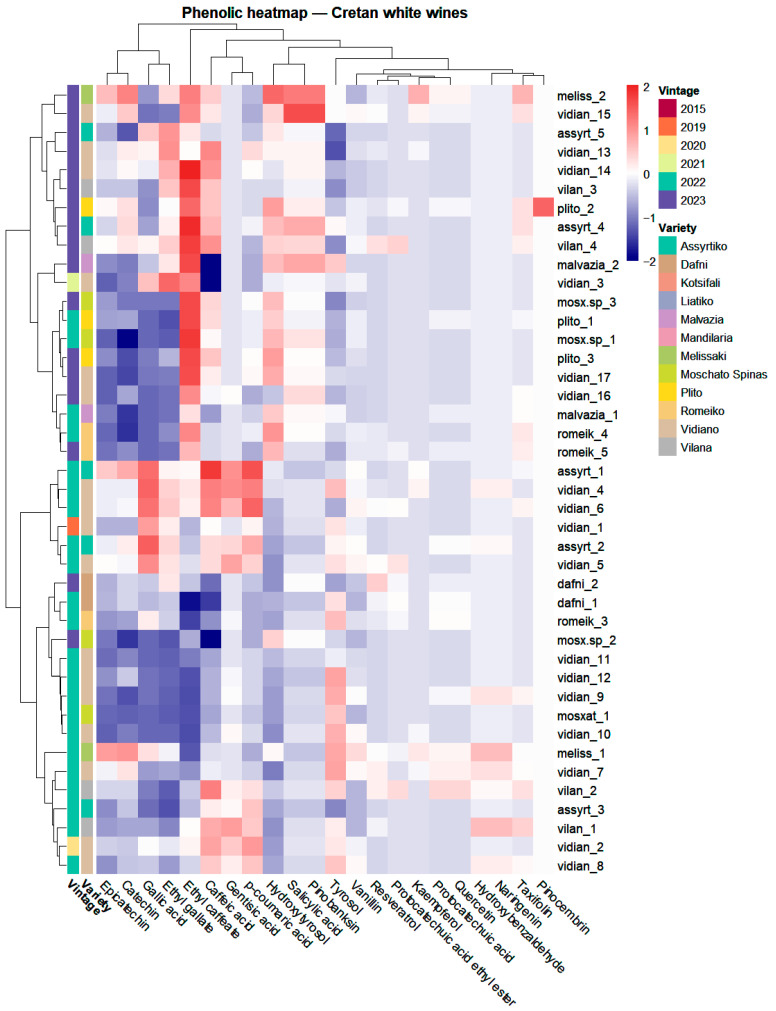
Hierarchical clustering heatmap of phenolic composition in Cretan white wine varieties.

Following the same unsupervised workflow applied to the red wines, the targeted phenolic dataset of Cretan white wines was primarily explored by HCA/heatmap visualization, while PCA was used as a complementary projection tool (Supplementary Figure S2a). Compounds absent across the dataset or detected only sporadically were removed to reduce sparsity; in the white-wine dataset, robust filtering was applied, considering only varieties represented by ≥3 wines. The resulting matrix was log-transformed and Pareto-scaled, and hierarchical clustering was performed using Euclidean distance and Ward’s linkage, with simultaneous clustering of samples and variables and heatmap annotation by variety and vintage (Figure 5).

In contrast to the red-wine dataset, where the unsupervised structure was partly aligned with variety, the white-wine heatmap showed limited variety-driven organization, with clusters frequently comprising multiple varieties and separation more often reflecting overall abundance gradients among the most consistently quantified phenolics (Figure 5). This pattern is consistent with literature indicating that wine polyphenol composition is shaped by a multifactorial interplay, extending beyond grape genetic background to include site- and climate-related effects, vintage, and oenological practices spanning pre-fermentative, fermentative, and post-fermentative stages [1,5]. In the present dataset (Table S3), these sources of variability are captured, as white wines were collected across three Cretan sub-regions (Heraklion, Chania, and Rethymno), from multiple producers per variety (notably Vidiano, the most represented variety, *n* = 17, produced by eight wineries distributed across all three sub-regions), and across multiple vintages (2019–2023). Consistent with this, same-variety wines from the same producer but different vintages did not systematically co-cluster, suggesting a non-negligible vintage contribution to the observed profiles.

At the variable level, the heatmap emphasized coordinated co-variation among a limited set of phenolics that dominated the structure of the matrix, most prominently flavan-3-ols (catechin, epicatechin) together with gallic acid and ethyl gallate, with secondary contributions from phenylethanol derivatives (e.g., tyrosol, hydroxytyrosol) and hydroxycinnamate-related compounds in subsets of wines. Overall, these co-varying modules contributed to the intensity-gradient appearance of the heatmap, rather than yielding clear, variety-specific blocks.

PCA performed on the same preprocessed matrix (varieties with ≥3 samples; Supplementary Method S4, Figure S2a) further supported the heatmap findings. The PCA diagnostics and cross-validation results, provided in Tables S11 and S12, confirm the robustness of the model. Although PC1 and PC2 together explained a large fraction of variance (PC1 = 41.2%, PC2 = 23.2%), the score plot showed substantial overlap among varieties and no consistent separation at the whole-profile level.

Taken together, HCA and PCA indicate that the targeted phenolic layer alone is insufficient for robust varietal discrimination across the Cretan white-wine dataset. This constraint is particularly pertinent for white wines, where limited skin/seed contact during vinification can reduce the abundance and dynamic range of several phenolic subclasses, thereby compressing the chemometric space available for variety-level separation. Accordingly, and in line with current wine-authentication frameworks, more reliable classification is generally achieved when phenolic fingerprints are complemented by orthogonal marker domains [63]. Most notably, HS-SPME-GC-MS volatile fingerprints provide distinct cultivar-related signatures and are routinely exploited in the varietal classification of white wines. Furthermore, broader metabolomics coverage (e.g., GC–MS and/or NMR), and, when needed, genetic tools can be integrated as each analytical layer captures different facets of varietal, geographic, and process-related variability [64,65,66]. Therefore, integrating HS-SPME-GC-MS volatile profiling with the targeted phenolic panel applied here is a logical next step to improve discrimination among the more overlapping white varieties.

PLS-DA performed on the preprocessed matrix of white wines (≥3 samples; Supplementary Figure S1b) reiterates the PCA results, but with more distinct separation between the varieties, especially for Assyrtiko, Moschato Spinas, and the other varieties. The cross-validation results, along with the confusion matrix for the final white-wine PLS-DA model, are detailed in Supplementary Method S4 (Tables S13 and S14). Additionally, the top VIP-ranked phenolic predictors for the white-wine model are listed in Table S15.

#### 2.3.3. Multiple Factor Analysis (MFA): Linking Phenolic Composition with Classical Indices

Building on the limited varietal organization observed in HCA/PCA, MFA was used here to test whether the targeted phenolic dataset co-varies with routine indices related to overall phenolic content and white-wine color development. The MFA integrated the targeted phenolic block with two classical variables available for the white-wine subset: total phenolics by Folin–Ciocalteu (TPC_Folin) and absorbance at 420 nm (Ox_420), a commonly used operational descriptor of color evolution in white wines [67].

In the compromise space (Supplementary Figure S2b), clustering remained weak at the variety level, consistent with the unsupervised results. However, the classical-variable projection showed a clear asymmetry: A_420_ aligned strongly with the first two MFA dimensions (Dim1 = 0.706; Dim2 = −0.392), whereas TPC exhibited a negligible projection on the same plane (Dim1 = 0.061; Dim2 = 0.028). This indicates that, within the first two MFA dimensions, variability in A420 color development is more concordant with the structured variation in targeted phenolics than is bulk Folin–Ciocalteu reactivity. The multivariate pattern is consistent with the univariate classical analysis, where A_420_ displayed the strongest varietal effect among the recorded indices (Welch *p* < 0.001; η^2^ = 0.768; Table S7). Given that vinification choices in white wines (e.g., maceration vs. non-maceration strategies) can substantially modulate phenolic extraction and associated color-related properties, this provides a plausible basis for why a color-evolution descriptor (Ox_420) may align with phenolic fingerprints even when stable varietal clustering is not recovered in a heterogeneous commercial dataset [68].

### 2.4. Phenolic Composition in Relation to Wine Quality and Bioactive Relevance

Wine phenolic compounds contribute to key dimensions of wine quality and typicity, including color development and evolution, oxidative behavior, and sensory attributes such as bitterness and astringency [2,69,70]. Astringency is a complex mouthfeel sensation arising from phenolic interactions in the oral environment, and its intensity and character depend on phenolic structure as well as matrix factors; therefore, compositional data should be interpreted as indicating sensory tendencies rather than deterministic outcomes [71,72,73,74]. From a biological standpoint, wine polyphenols are widely discussed in relation to antioxidant and anti-inflammatory mechanisms; however, mechanistic evidence for isolated compounds should not be translated into health claims for a given wine because physiological relevance depends strongly on dose, metabolism, and bioavailability [3,75,76,77].

Across the red cultivars, varietal differences in palate structure are expected to be governed largely by the balance of flavan-3-ols and related phenolics, which are consistently implicated as contributors to bitterness and astringency through their interactions with saliva and other wine-matrix components [70,71,72]. Beyond concentration, flavan-3-ol stereochemical composition can also be relevant: González-Muñoz et al. summarize evidence that a higher relative epicatechin contribution is associated with increased astringency intensity, whereas a higher relative catechin contribution is associated with decreased astringency [71]. Within this framework, Liatiko, characterized by comparatively elevated epicatechin together with flavonols (quercetin, kaempferol) and phenolic acids, is consistent with a wine style where a stronger phenolic contribution to bitterness/astringency is plausible, with perceived quality depending on whether this structure is balanced by acidity and overall flavor intensity [70,71,72]. From a compositional bioactive perspective, the flavonol signal is notable; quercetin has been reported to counteract inflammatory signaling by inhibiting NF-κB nuclear translocation and reducing TLR2/TLR4 expression [75,78]. The presence of resveratrol may likewise be highlighted as a compositional feature of interest; however, physiological relevance cannot be inferred from concentration alone given extensive metabolism and low systemic availability [41,62,75,79].

Kotsifali exhibits a flavan-3-ol–dominant phenolic pattern, with higher catechin than Liatiko and a substantial epicatechin contribution, supporting the potential for a firm phenolic palate structure. From a bioactive-composition perspective, Kotsifali’s representation of flavonoid subclasses such as naringenin (and pinobanksin) is best interpreted as expanding polyphenol diversity. Naringenin is reviewed as exhibiting multiple biological activities across in vitro and in vivo studies (including antidiabetic and anticancer effects), with mechanisms largely discussed in relation to antioxidant and anti-inflammatory pathways; however, optimal dosing and approaches to improve bioavailability remain insufficiently established [80].

Mandilaria, showing the highest mean catechin together with higher tyrosol, is the cultivar most plausibly aligned with a firmer phenolic taste/mouthfeel profile in this dataset. This inference is supported by two converging points: flavan-3-ols are repeatedly implicated in bitterness/astringency-related perception, and tyrosol has been described as capable of contributing bitterness at typical wine levels [69,71,72]. From an oenological perspective, the expression of these compounds is tightly linked to harvest decisions and vinification choices [70]. From a bioactive standpoint, Mandilaria’s enrichment in flavan-3-ols aligns with the broader red-wine polyphenol literature discussing modulation of endogenous antioxidant defenses and inflammatory signaling [75,81].

In contrast, Romeiko was distinguished by a hydroxycinnamate/phenylethanoid signature led by ethyl caffeate with accompanying p-coumaric acid and hydroxytyrosol, suggesting a different balance of phenolic drivers than the flavanol-forward reds. Hydroxycinnamic acids are described as slightly bitter with mild acidity and are often more relevant to oxidation chemistry than to strong direct taste effects, supporting the expectation of a comparatively softer phenolic-driven structure [70]. At the same time, hydroxycinnamate derivatives are relevant to wine aroma chemistry because p-coumaric and ferulic acid derivatives can act as precursors in pathways leading to volatile phenols under microbial processes [2,82]. From a bioactive perspective, Romeiko’s co-occurrence of caffeic-acid derivatives and hydroxytyrosol-type phenylethanoids fits within phenolic classes widely discussed for antioxidant and anti-inflammatory relevance [60,76,83]. Nevertheless, cultivar-level inference for Romeiko must remain cautious due to the limited subset (*n* = 3) and the presence of a 2015 vintage sample, which may reflect additional aging-related compositional variability beyond cultivar effects.

For the conventionally vinified white wines, weaker phenolic-driven mouthfeel effects are expected, not because phenolics are irrelevant, but because the phenolic pool is generally smaller and skewed toward low-molecular-weight compounds. Under these conditions, bitterness and texture differences can still emerge, yet they are more likely to reflect subtle shifts within a narrow set of phenolic families, strong matrix modulation (notably pH, ethanol, and residual sugar), and mixture effects that may be additive or non-linear [73,74]. Importantly, even this modest phenolic fraction can be biologically meaningful: white wines are commonly dominated by hydroxycinnamic acids and their tartaric esters (with caftaric acid often representing a major share) and may also contain yeast-derived metabolites such as tyrosol and hydroxytyrosol. Several authors have proposed that tyrosol and caffeic-acid–related constituents could contribute, at least in part, to the beneficial effects sometimes discussed for moderate white-wine consumption, with their relative relevance potentially heightened in white wines because the background levels of other phenolics are lower [76].

Within this framework, Vidiano provides the most stable reference among the conventionally vinified whites, spanning flavan-3-ols alongside the hydroxycinnamate/phenylethanoid space (ethyl caffeate; hydroxytyrosol). Consistent with the white-wine mouthfeel literature, this profile supports at most a modest phenolic contribution to bitterness/texture, with the sensory expression expected to be strongly conditioned by matrix factors and mixture behavior rather than by individual compounds alone [73]. Assyrtiko, expressing the most flavan-3-ol–forward pattern among these whites, is the cultivar most plausibly associated with a relative tendency toward higher bitterness within the white subset; however, mechanistic evidence summarized for white wines remains mixed regarding how strongly monomer/dimer phenolics contribute to astringency across different models, so any claim of pronounced astringency would be unsupported from phenolics alone [73]. For Vilana, the comparatively higher protocatechuic acid ethyl ester together with a relatively stronger stilbene (resveratrol) signal is more appropriately discussed as compositional interest for the wine polyphenol narrative than as a clear mouthfeel driver [76,84]. Plito and Malvazia, defined by strong ethyl caffeate/hydroxytyrosol or hydroxytyrosol signals, should similarly be interpreted with critical caution: the white-wine literature emphasizes that many hydroxycinnamate derivatives are often near or below individual sensory thresholds [73]. Finally, Melissaki was treated separately because its extended skin contact resulted in elevated flavan-3-ols and flavonols; accordingly, it is more likely to display increased bitterness and a firmer phenolic mouthfeel. From a bioactive-composition perspective, this enrichment is consistent with a higher phenolic antioxidant potential [75,76].

### 2.5. Limitations

This study represents a cross-sectional compilation of monovarietal Cretan wines collected across multiple wineries and vintages; therefore, the multivariate patterns should be interpreted as varietal signatures within this sampling frame rather than strict causal attribution exclusively to cultivar. Differences in viticultural conditions, vinification choices, terroir, and bottle aging may contribute to within-variety dispersion. As an exploratory sensitivity visualization, PCA score plots were additionally examined by winery code and vintage year (Figure S3). Winery labels were broadly interspersed, whereas vintage labeling suggested a modest trend mainly in the red-wine subset (including the presence of an older-vintage outlier), indicating that vintage/aging may modulate profiles without superseding cultivar-level structure. Finally, the limited sample size for certain white varieties (*n* = 2) restricts generalization, and these findings should be considered preliminary. Future work using more balanced designs (e.g., the same winery across multiple vintages and/or multiple wineries within the same vintage) and complementary volatile profiling is expected to strengthen attribution and improve discrimination among closely related cultivars.

## 3. Materials and Methods

### 3.1. Sample Collection

**Figure 6:**
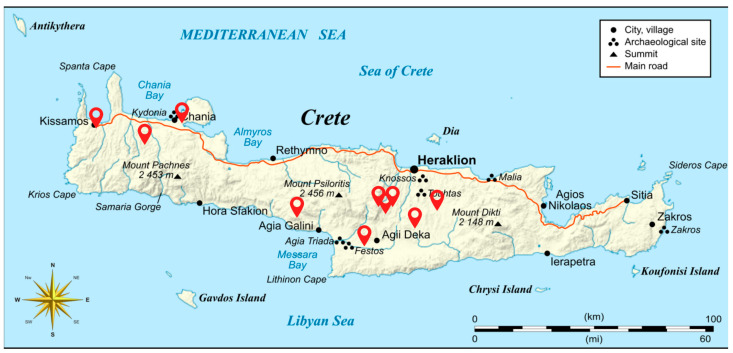
Locations of the wine samples collected from Crete. Adapted from “Crete integrated map-mk.svg” by Eric Gaba (Sting) (translated by MacedonianBoy), via Wikimedia Commons, CC BY-SA 4.0. Compass rose based on “Brújula.svg” by Serg!o. Changes: winery locations/markers [85].

A total of 67 monovarietal Cretan wines (42 white and 25 red; *Vitis vinifera* L.) were collected, belonging to the following indigenous grape varieties: Assyrtiko (*n* = 5), Dafni (*n* = 2), Vidiano (*n* = 17), Vilana (*n* = 4), Malvazia (*n* = 2), Melissaki (*n* = 2), Moschato Spinas (*n* = 4), Plito (*n* = 3), Romeiko (*n* = 6), Kotsifali (*n* = 6), Liatiko (*n* = 12), and Mandilaria (*n* = 4). Wines originated from ten wineries in Crete (Figure 6), specifically from the regions of Heraklion, Chania, and Rethymno, and covered mostly vintages 2022–2023 with only a few samples from previous years. Samples were either commercially available bottles or were bottled directly from winery tanks under anaerobic conditions, then stored at −18 °C until analysis. Prior to analysis, wines were thawed at 4 °C and homogenized. Detailed information on sample origin, variety, region, vintage year, and winery code is provided in Table S16 (Supplementary Material). To explicitly summarize the distribution of wineries and vintages within each variety, a cross-tabulation of variety × winery code × vintage year with sample counts (*n*) is provided in Table S16.

### 3.2. Classical Oenological Analyses

Routine oenological parameters and spectrophotometric indices (free/total SO_2_, titratable acidity, pH, tannins, phenolic content index at 280 nm, total phenolics by Folin–Ciocalteu, reducing sugars, color intensity, and hue) were determined using standardized procedures (OIV methods where applicable). Full details on reagents, sample handling, and methodology are provided in Supplementary Material (Method S5).

### 3.3. Phenolic Profiling Using LC-QTOF/MS

Determination of phenolic compounds was performed using liquid chromatography coupled to quadrupole time-of-flight mass spectrometry (LC–QTOF–MS), following a previously validated protocol for wine metabolomics [29,38].

Sample preparation. Bottles were uncorked while applying a gentle nitrogen flush at the bottle headspace to minimize oxygen exposure during opening and subsampling. Immediately after opening, 3 mL aliquots of each wine were filtered through 0.22 μm regenerated cellulose syringe filters (Phenomenex, Torrance, CA, USA). Filtered samples (990 μL) were spiked with 10 μL of an internal standard (ethyl vanillin, 2 mg·L^−1^ final concentration) and transferred to amber autosampler vials.

Quality control. A pooled quality control (QC) sample was prepared by combining equal aliquots from all wines, thus representing their weighted average composition. The QC was injected six times before the analytical sequence to stabilize the system and assess performance and was subsequently reanalyzed at regular intervals (every ten injections) throughout the batch to monitor instrumental stability, retention time reproducibility, and signal intensity drift.

Chromatographic separation. Analysis was carried out on a Dionex UltiMate 3000 RSLC system (Thermo Fisher Scientific, Dreieich, Germany) equipped with an Acclaim RSLC 120 C18 column (2.1 × 100 mm, 2.2 μm; Thermo Fisher Scientific) and a VanGuard BEH C18 pre-column (Waters). The column was maintained at 30 °C. The mobile phases were: (A) 5 mM ammonium acetate in water/methanol (90:10, *v*/*v*) and (B) 5 mM ammonium acetate in methanol. The gradient was: 1% B (0–1 min, 0.2 mL/min), 1–39% B (1–4 min, 0.2 mL/min), 39–99.9% B (4–15 min, 0.4 mL/min), held at 99.9% B (15–17 min, 0.48 mL/min), and re-equilibrated at initial conditions for 3 min (0.2 mL/min). Injection volume was 5 μL.

Mass spectrometry. The LC was coupled via an electrospray ionization (ESI) source, operated in negative ion mode, to a Maxis Impact QTOF mass spectrometer (Bruker Daltonics, Bremen, Germany). Ionization parameters were set as follows: capillary voltage 3.5 kV, end plate offset −500 V, nebulizer 2.0 bar (N_2_), dry gas 8 L/min, dry temperature 200 °C. Mass spectra were acquired over *m*/*z* 50–1000 at 2 Hz. All wine samples were analyzed using broadband collision-induced dissociation (bbCID, data-independent acquisition), which alternates between low (4 eV) and high (25 eV) collision energies to simultaneously record MS and MS/MS spectra.

Calibration. External calibration was performed daily using a sodium formate solution (10 mM in water/isopropanol, 1:1, *v*/*v*). Internal calibration was automatically applied at the beginning of each run (0.1–0.25 min).

Data Analysis. For the determination of phenolic compounds, a target screening workflow was applied using an in-house database comprising 45 analytes from different phenolic subclasses (Table S17, Supplementary Material). For each target analyte, the database contained the molecular formula, the exact *m*/*z* of the [M-H]^−^ precursor ion, the experimentally determined retention time (t_R_, min), and characteristic MS/MS product ions (Table S17, Supplementary Material). After data acquisition, raw files were processed with DataAnalysis 4.4 and TASQ 1.4 (Bruker Daltonics, Bremen, Germany). Identification and quantification were performed using authentic reference standards analyzed in the same batch and on the same day as the samples, following the workflow described by Dasenaki et al. (2019) [86]. Target compounds were accepted as positively identified only when they met all of the following criteria: mass accuracy < 2 mDa, retention time deviation < 0.2 min relative to the corresponding standard, isotopic pattern fit (mSigma) ≤ 50, peak area > 1000, peak intensity > 500, and agreement of the MS/MS fragmentation pattern with that of the reference standard. Extracted ion chromatograms (EICs) of the precursor ions were generated for all targets meeting the peak area and intensity thresholds and were visually inspected across all samples.

Preparation of standard solutions and calibration curves. Individual standard stock solutions were prepared in methanol (MeOH) at 1000 μg·mL^−1^ and stored at −20 °C in amber glass bottles. Mixed working standard solutions containing all target analytes were prepared by serial dilution of the stock solutions in MeOH/water (1:1, *v*/*v*) to construct external calibration curves. External calibration curves were constructed for all target analytes over the concentration range 0.1–20 mg·L^−1^. Samples with concentrations exceeding the upper limit of the calibration range were appropriately diluted and re-analyzed. Conversely, analytes present at levels below their respective compound-specific limits of quantification (LOQs) were reported as <LOQ to ensure analytical reliability, in accordance with previously validated methods [29,38]. The corresponding coefficients of determination (R^2^) are reported in Table S18 (Supplementary Material).

### 3.4. Statistical Analysis

#### 3.4.1. Univariate Statistical Analysis

For the target phenolic compounds, concentrations falling below the limit of quantification (<LOQ) were replaced with half of the respective LOQ (LOQ/2) prior to data analysis. For all classical oenological parameters and target phenolic compounds, descriptive statistics (mean, standard deviation, minimum, and maximum) were calculated separately for each grape variety. Differences among varieties were evaluated using one-way ANOVA with grape variety as a fixed factor (IBM SPSS Statistics, v.30.0; IBM Corp., Armonk, NY, USA). Approximate normality of residuals was examined with Shapiro–Wilk tests and inspection of Q–Q plots. Homogeneity of variances was assessed with Levene and Brown–Forsythe tests. As the numbers of wines per variety were unequal, and several variables showed variance heterogeneity, we used Welch’s heteroscedastic F-test as the primary omnibus test instead of the classical one-way ANOVA F-test; simulation studies have shown that Welch’s ANOVA provides better control of Type I error and power than the classical F-test or Brown–Forsythe ANOVA under unequal variances and unbalanced group sizes. Brown–Forsythe-type procedures were retained as robust diagnostics for the homogeneity-of-variance assumption [87,88]. When the Welch ANOVA indicated significant overall differences (*p* < 0.05), pairwise comparisons between grape varieties were performed using the Games–Howell post hoc test (equal variances not assumed). Statistical significance for all univariate tests was set at *p* < 0.05. Distributional patterns and potential outliers for each phenolic compound were further assessed using variety-stratified box-and-whisker plots for red and white wines, generated in R (ggplot2) from the raw concentration data.

#### 3.4.2. Multivariate Statistical Analysis

Multivariate exploration of the wine phenolic profiles was performed in R (version 4.5.0) (R Foundation for Statistical Computing, Vienna, Austria) within RStudio (version 2025.09.2+418]. Concentrations reported as “ND” or equal to zero were treated as missing values. Phenolic variables with more than 20% missing entries were discarded, and wines with no remaining non-missing phenolic measurements were removed. For the retained variables, missing values were imputed using a minimum-value approach (impute_min, imputomics package), after which data were transformed using log_1_p(x) and Pareto-scaled (centering and division by the square root of the standard deviation of each variable).

To integrate targeted phenolic fingerprints with routine compositional measurements, we a priori selected classical oenological indices that are chemically or spectrophotometrically related to phenolic content and phenolic-driven optical characteristics. Classical oenological parameters (phenolic content index at 280 nm, tannins, absorbance at 420 nm, color intensity, color hue, and total phenolic content by Folin–Ciocalteu) were averaged across replicate measurements for each wine and standardized (z-scores). These indices were then merged with the processed phenolic concentration dataset via the common sample identifier to construct a two-block dataset. Multiple Factor Analysis (MFA) was carried out with the FactoMineR package, treating phenolics and classical indices as separate quantitative groups. A comprehensive MFA model was initially constructed using the full set of wines, after which separate MFA models were estimated for (i) red wines, incorporating phenolic variables together with PCI, tannins, and Folin–Ciocalteu TPC, and (ii) white wines, incorporating phenolic variables together with Folin–Ciocalteu TPC only, reflecting the completeness of the available classical indices for each color group. Individual maps with 95% confidence ellipses by grape variety, group representations, and vectors for the classical indices were visualized using FactoMineR, factoextra, and ggplot2.

Principal Component Analysis (PCA) was then applied to the log-transformed, Pareto-scaled phenolic dataset (prcomp function) to summarize the main sources of variance in phenolic composition. Separate PCA models were constructed for (i) all wines combined, (ii) red wines only, and (iii) white varieties represented by at least three samples. For each of these blocks, the suitability of the correlation matrix for PCA was checked with the Kaiser–Meyer–Olkin (KMO) measure and Bartlett’s test of sphericity (psych package). The number of components retained for interpretation was guided by scree plots, parallel analysis, and cross-validated reconstruction performance (Supplementary Methods S1 and S3). Score plots colored by wine color or variety, together with loading plots for phenolic variables, were used to assess clustering patterns and to identify compounds contributing most strongly to the differentiation of Cretan wine varieties.

In addition, supervised classification was performed using partial least squares–discriminant analysis (PLS-DA) implemented in mixOmics, with grape variety as the response and the processed dataset of quantified phenolic variables as predictors. Separate PLS-DA models were built for red wines (all red varieties) and for white wines (retaining only varieties with ≥3 samples). Model complexity was selected by repeated M-fold cross-validation (five folds, 50 repeats) by minimizing the balanced error rate (BER), and variable importance was summarized using VIP scores. Full modelling parameters and outputs are provided in the Supplementary Material (Methods S2 and S4).

## 4. Conclusions

The present study uses targeted LC–QTOF–MS profiling across 12 monovarietal Cretan cultivars to expand the phenolic evidence base for indigenous Greek wines, providing first-time targeted profiles for Dafni, Plito, Moschato Spinas, Malvazia, Vilana, Melissaki, and Romeiko under both red- and white-style vinification. This work significantly expands the existing phenolic evidence base for Greek wines, contributing novel insights into the chemical composition of these underrepresented cultivars. Such advances will enhance varietal classification, contribute to the authenticity assessment of Greek wines, and promote the characterization of quality markers that support the global competitiveness of Greek wines. Furthermore, this study provides valuable data for researchers, producers, and regulatory mechanisms aiming to ensure the authenticity and high quality of Cretan wines, aligning with both national and international standards for wine production.

Overall, the dataset reveals chemically coherent phenolic patterns. However, phenolic-only PCA and PLS-DA did not provide robust within-subset discrimination among red and white varieties, with Mandilaria showing the most consistent separation. Furthermore, given the restricted sample availability for certain rare cultivars, specifically Dafni, Malvazia, and Melissaki (*n* = 2); Plito (*n* = 3); Moschato Spinas (*n* = 4); and Romeiko (*n* = 3 for both red and white vinifications), the specific phenolic signatures reported for these varieties must be considered preliminary. These findings highlight the need for further research, including increased per-variety representation across multiple vintages, as well as the integration of complementary chemical layers, such as volatile profiling and broader metabolomics.

## Figures and Tables

**Table 1:** Concentrations of target compounds detected in the red wine samples (mg·L^−1^).

Flavonoid/Non-Flavonoid	Phenolic Category	Compounds	Kotsifali (*n* = 6)	Liatiko (*n* = 12)	Mandilaria (*n* = 4)	Romeiko (*n* = 3)	Welch *p*	η^2^
Flavonoid	Flavan-3-ol (flavanol)	Catechin	15.01 ± 2.98 ^a^	12.39 ± 5.58 ^ab^	19.41 ± 5.30 ^ab^	8.56 ± 1.02 ^b^	0.003	0.337
		Epicatechin	7.29 ± 1.77 ^a^	8.11 ± 3.47 ^a^	7.10 ± 1.33 ^a^	3.66 ± 0.32 ^b^	<0.001	0.236
	Flavanone	Eriodictyol	<LOQ	<LOQ	<LOQ	<LOQ	.	.
		Naringenin	15.01 ± 6.04	11.82 ± 4.98	9.82 ± 3.36	10.43 ± 1.42	0.384	0.141
		Pinocembrin	<LOQ	<LOQ	0.14 (*n* = 1)	<LOQ	.	.
		Sakuranetin	0.17 (*n* = 1)	N.D.	3.18 (*n* = 1)	N.D.	.	.
	Flavanonol	Pinobanksin	14.84 ± 5.27	12.58 ± 5.35	11.08 ± 5.26	11.72 ± 2.73	0.699	0.069
		Taxifolin	0.17 ± 0.10	0.14 ± 0.08	0.09 ± 0.04	0.30 ± 0.18	0.191	0.319
		Chrysin	<LOQ	N.D.	<LOQ	N.D.	.	.
		Luteolin	0.16 ± 0.11	0.12 ± 0.13	<LOQ	0.12 (*n* = 1)	.	0.074
		Kaempferol	0.29 ± 0.10 ^ab^	0.32 ± 0.13 ^b^	0.15 ± 0.06 ^a^	0.12 ± 0.09 ^ab^	0.031	0.357
		Myricetin	0.19 ± 0.13	0.13 ± 0.08	0.37 ± 0.18	0.16 ± 0.15	0.213	0.390
		Quercetin	3.13 ± 0.69	2.97 ± 1.69	1.89 ± 1.04	0.72 ± 1.16	0.068	0.286
Non-flavonoid	Benzoic acid	Gallic acid	8.60 ± 5.96 ^ab^	9.90 ± 7.06 ^a^	7.54 ± 0.49 ^ab^	2.11 ± 1.71 ^b^	0.01	0.168
		Gentisic acid	0.18 ± 0.13	0.09 ± 0.04	0.13 ± 0.03	0.15 ± 0.10	0.187	0.241
		Protocatechuic acid	0.43 ± 0.36 ^a^	0.33 ± 0.15 ^a^	0.34 ± 0.08 ^a^	0.22 ± 0.01 ^a^	0.037	0.094
		Salicylic acid	0.27 ± 0.15	0.42 ± 0.58	0.69 ± 0.76	0.15 ± 0.02	0.156	0.101
		Syringic acid	<LOQ	0.33 (*n* = 1) ^a^	0.33 ± 0.09 ^a^	0.22 (*n* = 1) ^b^	0.004	0.666
		Vanillic acid	N.D.	0.18 (*n* = 1)	0.74 (*n* = 1)	N.D.	.	.
		3-hydroxybenzoic acid	0.35 ± 0.19	0.44 ± 0.34	0.50 ± 0.05	0.30 ± 0.16	0.24	0.073
		4-hydroxybenzoic acid	0.17 ± 0.15	0.12 ± 0.07	N.D.	N.D.	0.721	0.065
	Benzoic acid derivative	Ethyl gallate	5.28 ± 2.65	5.67 ± 2.33	7.76 ± 0.74	3.13 ± 2.98	0.051	0.248
		Protocatechuic acid ethyl ester	0.46 ± 0.29 ^ab^	0.29 ± 0.11 ^b^	0.08 ± 0.02 ^a^	0.20 ± 0.05 ^ab^	0.001	.
	Benzoic aldehyde	Hydroxybenzaldehyde	<LOQ	0.17 ± 0.10	<LOQ	<LOQ	.	0.33
		Syringaldehyde	0.10 (*n* = 1)	0.44 ± 0.33	0.21 (*n* = 1)	0.20 ± 0.20	.	0.181
		Vanillin	0.55 ± 0.24	0.53 ± 0.27	1.26 ± 0.42	0.53 ± 0.38	0.113	0.482
		Ethyl caffeate	5.14 ± 4.09	16.01 ± 16.93	2.70 ± 3.78	35.45 ± 55.33	0.195	0.204
	Hydroxycinnamic acid	Caffeic acid	2.50 ± 0.75 ^b^	5.48 ± 2.64 ^a^	3.43 ± 0.88 ^ab^	5.09 ± 2.13 ^ab^	0.045	0.312
		p-coumaric acid	0.57 ± 0.57	1.01 ± 0.99	0.36 ± 0.16	1.32 ± 0.92	0.167	0.147
		Cinnamic acid	2.12 ± 0.94	0.72 ± 0.49	6.99 (*n* = 1)	0.35 (*n* = 1)	.	0.932
	Phenolic acid derivative	Homovanillic acid	1.32 ± 1.97	0.68 ± 0.91	0.70 ± 1.08	0.21 ± 0.19	0.477	0.101
	Phenylethanol derivativ	Hydroxytyrosol	1.56 ± 1.33	1.67 ± 1.71	2.39 ± 2.57	2.50 ± 2.75	0.916	0.042
		Tyrosol	17.11 ± 5.89 ^b^	14.61 ± 6.94 ^b^	33.64 ± 5.97 ^a^	19.54 ± 15.98 ^ab^	0.015	0.457
	Simple phenol	Catechol	<LOQ	<LOQ	<LOQ	<LOQ	.	0.136
	Stilbene	Resveratrol	0.45 ± 0.29	0.52 ± 0.45	0.23 ± 0.09	0.39 ± 0.08	0.097	0.085

N.D.: not detected. Values are reported as mean ± SD. Different superscript letters within the same row (i.e., for the same compound) indicate statistically significant differences among wine varieties (Welch’s ANOVA followed by Games–Howell post hoc test, *p* < 0.05). Means sharing at least one letter are not significantly different (e.g., ab does not differ from a or b).

**Table 2:** Concentrations of target compounds detected in the white wine samples (mg·L^−1^).

Flavonoid/Non-Flavonoid	PhenoLic Category	Compound	Vidiano (*n* = 17)	Vilana (*n* = 4)	Moschato Spinas (*n* = 4)	Assyrtiko (*n* = 5)	Plito (*n* = 3)	Romeiko (*n* = 3)	Dafni (*n* = 2)	Malvazia (*n* = 2)	Melissaki (*n* = 2)	Welch *p*	η^2^
Flavonoid	Flavan-3-ol (flava-nol)	Catechin	3.75 ± 2.35 ^a^	4.01 ± 1.99 ^ab^	0.74 ± 0.57 ^b^	6.08 ± 4.34 ^ab^	3.49 ± 3.48 ^ab^	1.50 ± 0.91 ^ab^	3.63 ± 0.06 ^a^	1.00 ± 0.64 ^ab^	15.91 ± 2.03 ^ab^	<0.001	0.651
		Epicatechin	1.02 ± 0.81 ^a^	1.32 ± 0.72 ^a^	0.30 ± 0.26 ^a^	1.68 ± 1.41 ^a^	1.15 ± 1.09 ^a^	0.26 ± 0.23 ^a^	0.85 ± 0.09 ^a^	0.32 ± 0.10 ^a^	5.47 ± 1.21 ^a^	0.01	0.659
	Flavanone	Eriodictyol	<LOQ	<LOQ	<LOQ	<LOQ	0.12 ± 0.13	<LOQ	<LOQ	<LOQ	<LOQ	.	.
		Hesperetin	<LOQ	N.D.	N.D.	<LOQ	N.D.	N.D.	N.D.	N.D.	N.D.	.	.
		Naringenin	19.33 ± 18.23	8.16 ± 7.63	3.53 ± 2.84	21.06 ± 30.59	34.09 ± 38.42	6.75 ± 2.85	7.52 ± 3.15	10.34 ± 5.44	10.69 ± 3.88	0.43	0.169
		Pinocembrin	<LOQ	<LOQ	<LOQ	<LOQ	0.13 ± 0.22	<LOQ	<LOQ	<LOQ	<LOQ	.	.
		Sakuranetin	0.80 ± 0.41	0.40 (*n* = 1)	N.D.	1.47 (*n* = 1)	11.45 (*n* = 1)	N.D.	N.D.	N.D.	0.17 (*n* = 1)	.	0.998
	Flavanonol	Pinobanksin	19.59 ± 18.11	8.61 ± 7.91	3.96 ± 2.51	22.24 ± 30.59	24.16 ± 32.94	7.04 ± 2.92	8.18 ± 3.84	10.98 ± 5.78	10.77 ± 3.17	0.313	0.140
		Taxifolin	<LOQ	0.17 ± 0.09	<LOQ	0.12 ± 0.07	0.13 ± 0.09	0.13 ± 0.10	<LOQ	0.10 ± 0.05	0.24 ± 0.17	.	0.350
	Flavone	Chrysin	<LOQ	<LOQ	N.D.	<LOQ	<LOQ	N.D.	N.D.	N.D.	<LOQ	.	.
		Luteolin	<LOQ	<LOQ	N.D.	<LOQ	N.D.	N.D.	<LOQ	N.D.	0.34 (*n* = 1)	.	0.477
	Flavonol	Galangin	<LOQ	N.D.	N.D.	<LOQ	N.D.	N.D.	<LOQ	N.D.	<LOQ	.	.
		Kaempferol	<LOQ	<LOQ	<LOQ	<LOQ	<LOQ	<LOQ	<LOQ	<LOQ	<LOQ	.	.
		Quercetin	0.16 ± 0.15	<LOQ	<LOQ	0.72 ± 0.70	<LOQ	<LOQ	0.10 ± 0.07	<LOQ	1.15 ± 0.70	0.062	0.311
Non-flavonoid	Benzoic acid	3-hydroxybenzoic acid	0.29 ± 0.18	0.51 (*n* = 1)	N.D.	<LOQ	<LOQ	<LOQ	0.32 (*n* = 1)	N.D.	0.13 (*n* = 1)	.	0.166
		4-hydroxybenzoic acid	<LOQ	<LOQ	N.D.	N.D.	0.09 (*n* = 1)	N.D.	N.D.	<LOQ	<LOQ	.	.
		Gallic acid	3.64 ± 4.60	1.09 ± 1.24	0.12 ± 0.09	6.49 ± 6.68	0.25 ± 0.17	3.22 (*n* = 1)	1.14 ± 0.16	1.19 (*n* = 1)	2.14 ± 2.32	.	0.208
		Gentisic acid	0.17 ± 0.17	0.20 ± 0.21	<LOQ	0.21 ± 0.22	<LOQ	<LOQ	<LOQ	<LOQ	<LOQ	.	0.177
		Protocatechuic acid	0.10 ± 0.05	0.22 ± 0.19	<LOQ	0.12 ± 0.04	0.11 ± 0.04	0.13 ± 0.04	0.13 ± 0.04	0.11 (*n* = 1)	0.22 ± 0.00	.	0.311
		Salicylic acid	0.32 ± 0.43	0.29 ± 0.20	0.30 ± 0.16	0.30 ± 0.36	0.34 ± 0.10	0.22 ± 0.09	0.19 ± 0.16	0.65 ± 0.43	0.74 ± 0.91	0.906	0.116
	Benzoic acid derivative	Ethyl gal-late	2.53 ± 2.99	2.33 ± 2.33	0.17 ± 0.11	3.33 ± 2.57	1.15 ± 1.14	0.76 ± 0.60	2.29 ± 1.31	1.75 ± 2.04	2.89 ± 1.02	0.092	0.153
		Protocatechuic acid ethyl ester	<LOQ	0.17 ± 0.17	<LOQ	<LOQ	<LOQ	0.11 ± 0.01	0.14 ± 0.03	<LOQ	0.10 ± 0.01	.	0.329
	Benzoic aldehyde	Hydroxy-benzaldehyde	<LOQ	0.10 ± 0.13	<LOQ	<LOQ	<LOQ	<LOQ	<LOQ	<LOQ	0.15 ± 0.17	.	0.253
		Syringaldehyde	0.21 ± 0.06	N.D.	N.D.	0.48 (*n* = 1)	N.D.	0.46 (*n* = 1)	0.38 ± 0.09	N.D.	0.43 (*n* = 1)	.	0.872
		Vanillin	0.30 ± 0.11	<LOQ	<LOQ	0.31 ± 0.10	0.26 ± 0.03	0.25 ± 0.03	<LOQ	<LOQ	0.31 ± 0.41	.	0.263
	Hydroxycin-namate ester	Ethyl caffeate	22.58 ± 31.16 ^b^	44.94 ± 42.40 ^ab^	43.58 ± 47.23 ^ab^	28.02 ± 42.95 ^ab^	69.97 ± 12.45 ^a^	21.34 ± 19.32 ^ab^	3.02 ± 3.72 ^b^	45.99 ± 43.96 ^ab^	23.20 ± 30.73 ^ab^	0.013	0.198
	Hydroxycinnamic	Caffeic acid	6.00 ± 3.62	9.37 ± 2.26	3.07 ± 2.63	8.24 ± 6.75	6.41 ± 0.71	2.56 ± 0.82	<LOQ	1.73 (*n* = 1)	4.71 ± 2.24	.	0.334
		Cinnamic acid	0.50 ± 0.18	0.25 ± 0.14	0.52 ± 0.38	0.51 ± 0.17	0.54 ± 0.20	0.55 ± 0.08	1.20 (*n* = 1)	0.47 ± 0.59	3.27 (*n* = 1)	.	0.889
		Ferulic acid	N.D.	N.D.	N.D.	<LOQ	N.D.	N.D.	N.D.	<LOQ	N.D.	.	.
		p-coumaric acid	0.81 ± 0.83	0.63 ± 0.50	0.31 ± 0.26	1.35 ± 1.20	0.46 ± 0.14	0.19 ± 0.24	<LOQ	0.16 ± 0.12	<LOQ	0.099	0.242
	Phenolic acid derivative	Homovanillic acid	1.13 ± 0.94	2.07 ± 0.24	1.61 ± 0.07	1.18 ± 1.11	2.98 ± 0.77	1.38 ± 1.16	3.84 (*n* = 1)	1.48 ± 0.04	0.28 ± 0.17	.	0.549
	Phenylethanol derivative	Hydroxytyrosol	0.72 ± 0.73 ^a^	0.88 ± 0.76 ^ab^	1.80 ± 1.07 ^ab^	1.11 ± 0.92 ^ab^	2.82 ± 0.64 ^ab^	2.10 ± 1.64 ^ab^	0.30 ± 0.16 ^a^	2.26 ± 0.21 ^b^	3.14 ± 2.60 ^ab^	.	0.475
		Tyrosol	16.98 ± 6.84	13.00 ± 6.30	12.35 ± 6.27	11.32 ± 4.45	9.94 ± 0.56	14.62 ± 7.17	13.47 ± 6.77	17.79 ± 4.92	20.53 ± 8.57	0.214	0.204
	Simple phenol	Catechol	<LOQ	<LOQ	<LOQ	<LOQ	<LOQ	<LOQ	<LOQ	<LOQ	<LOQ	.	.
	Stilbene	Resveratrol	0.10 ± 0.08	0.23 ± 0.15	<LOQ	<LOQ	<LOQ	0.12 ± 0.01	0.32 ± 0.24	<LOQ	0.16 ± 0.05	.	0.456

N.D.: not detected. Values are reported as mean ± SD. Different superscript letters within the same row (i.e., for the same compound) indicate statistically significant differences among wine varieties (Welch’s ANOVA followed by Games–Howell post hoc test, *p* < 0.05). Means sharing at least one letter are not significantly different (e.g., ab does not differ from a or b).

## Data Availability

All data are contained within the article.

## Supplementary Materials

The following supporting information can be downloaded at: https://www.mdpi.com/article/10.3390/molecules31050815/s1, Figure S1: PLS-DA score plots based on the targeted phenolic composition (LC–QTOF-MS) of Cretan wines. (a) Cretan red wine varieties (Kotsifali, Liatiko, Mandilaria, Romeiko); (b) Cretan white wine varieties including only classes with ≥3 samples (Assyrtiko, Moschato Spinas, Plito, Romeiko, Vidiano, Vilana); Figure S2: Multivariate analysis of Cretan white wines by variety: (a) PCA score plot based on phenolic composition for varieties with ≥4 wines, with 95% confidence ellipses for Assyrtiko, Moschato Spinas, Vidiano, and Vilana; (b) MFA compromise map integrating the targeted phenolic block with classical indices (total phenolics by Folin–Ciocalteu, TPC_Folin, and absorbance at 420 nm, A420/Ox_420); Figure S3: PCA sensitivity plots colored by vintage year and winery code. (a,b) Red wines; (c,d) white wines (varieties with ≥3 samples per variety). Colors indicate vintage year (a,c) or winery code (b,d), while shapes indicate variety. The variance explained by PC1 and PC2 is reported on the axes; Table S1: Cretan PDO and PGI Regions: Classification, Registration, and Recognition Dates; Table S2: Recommended, permitted, and temporarily permitted grape varieties for Crete according to Ministerial Decision 247771/04-03-2010; Table S3: Classical oenological parameters of Cretan red wines by variety (mean ± SD); Table S4: Classical oenological parameters of Cretan white wines by variety (mean ± SD). Table S5: Variance explained by PCA components (red wines); Table S6: Variance explained by PCA components (red wines); Table S7: Cross-validated BER by component (red wines); Table S8: Confusion matrix for the final red-wine PLS-DA model (in-sample prediction; descriptive); Table S9: Top VIP-ranked phenolic predictors in the final red-wine PLS-DA model (selected component); Table S10: Concentrations (mg·L-1) of target compounds in Cretan white wines and univariate statistics (Welch’s ANOVA and η2) calculated after excluding Melissaki; Table S11: Variance explained by PCA components (white wines; ≥3 wines/variety); Table S12: Cross-validated Q2 (reconstruction) by number of PCs (white wines; ≥3 wines/variety); Table S13: Cross-validated BER by component (white wines); Table S14: Confusion matrix for the final white-wine PLS-DA model (in-sample prediction; descriptive); Table S15: Top VIP-ranked phenolic predictors in the final white-wine PLS-DA model (selected component); Table S16: Wine samples analyzed in the study: variety, color, winery code, vintage year, region, *n* (number of wine samples), and sample ID; Table S17: Target-screening database including precursor ion exact mass, retention time, and fragment ions; Table S18: R2 values of calibration curves for all quantified analytes.

## Abbreviations

The following abbreviations are used in this manuscript:

A_280_	Absorbance at 280 nm (phenolic content index; PCI)
A_420_	Absorbance at 420 nm
ANOVA	Analysis of variance
AutoMS	Automated MS/MS acquisition mode (data-dependent acquisition; Bruker)
bbCID	Broadband collision-induced dissociation (data-independent acquisition)
BEH	Bridged ethyl hybrid (stationary phase)
BER	Balanced error rate
CI	Color intensity
EICs	Extracted ion chromatograms
ESI	Electrospray ionization
FT-IR	Fourier-transform infrared spectroscopy
FWHM	Full width at half maximum
GAE	Gallic acid equivalents
GC–MS	Gas chromatography–mass spectrometry
HCA	Hierarchical clustering analysis
HPLC	High-performance liquid chromatography
I	Color intensity (index)
KMO	Kaiser–Meyer–Olkin measure
LC	Liquid chromatography
LC–MS	Liquid chromatography–mass spectrometry
LC–QTOF–MS	Liquid chromatography–quadrupole time-of-flight mass spectrometry
MFA	Multiple factor analysis
MS	Mass spectrometry
MS/MS	Tandem mass spectrometry
mSigma	Isotopic pattern fit score (Bruker)
*m*/*z*	Mass-to-charge ratio
N.D.	Not detected
N_2_	Nitrogen gas
NMR	Nuclear magnetic resonance
OIV	International Organisation of Vine and Wine
Ox_420	Absorbance at 420 nm used as oxidation/colour evolution index variable
PCA	Principal component analysis
PCI	Phenolic content index (A280)
PLS-DA	Partial least squares–discriminant analysis
QC	Quality control
QTOF	Quadrupole time-of-flight
RSLC	Rapid Separation Liquid Chromatography
SD	Standard deviation
SO_2_	Sulfur dioxide
SPSS	Statistical Package for the Social Sciences
T	Color hue (index)
TASQ	Target Analysis for Screening and Quantitation (Bruker software)
t_R_	Retention time
TPC	Total phenolic content
TPC_Folin	Total phenolic content by Folin–Ciocalteu
UHPLC	Ultra-high-performance liquid chromatography
VIP	Variable importance in projection
*v*/*v*	Volume/volume
η^2^	Eta-squared (effect size)
