# Supplementary material for: Decoding Cretan Wines: Phenolic Profiling of Greek Indigenous Wine Varieties Using LC-QTOF-MS
Source: Molecules. 2026 Feb 28;31(5):815. doi: 10.3390/molecules31050815 (PMC12986486; doi:10.3390/molecules31050815)
Supplement: Supplementary file 1 [file molecules-31-00815-s001.zip › molecules-4160274-supplementary.pdf]

Supplementary Materials

Table S1. Cretan PDO and PGI Regions: Classification, Registration, and Recognition Dates.

A/A	Region Name	Designation	Registration Code	Date of Recognition
1	Crete	PGI	PGI-GR-A1605	2011-12-20
2	Rethymno	PGI	PGI-GR-A1060	2011-12-20
3	Heraklion	PGI	PGI-GR-A1565	2009-08-01
4	Kissamos	PGI	PGI-GR-A0422	2009-08-01
5	Lasithi	PGI	PGI-GR-A0830	2009-08-08
6	Chania	PGI	PGI-GR-A1059	2011-12-20
7	Sitia	PDO	PDO-GR-A1608	1993-06-09
8	Peza	PDO	PDO-GR-A1531	1971-08-23
9	Daphnes	PDO	PDO-GR-A1533	1971-08-23
10	Archanes	PDO	PDO-GR-A1532	1971-08-23
11	Malvasia Handakas-Candia	PDO	PDO-GR-A1617	2011-06-16
12	Handakas-Candia	PDO	PDO-GR-A1615	2011-06-16

Table S2. Recommended, permitted and temporarily permitted grape varieties for Crete according to Ministerial Decision 247771/04-03-2010.

A/A	Category	Color	Variety	Synonyms
1	**Recommended**	Red	Thrapsa	–
2			Kotsifali	–
3			Ladikino	–
4			Liatiko	–
5			Mandilaria	Amorgiano; Doubraina Mavri; Kountoura Mavri
6			Cabernet Sauvignon	–
7			Carignan	–
8			Grenache Rouge	–
9			Syrah	–
10		White	Athiri	–
11			Assyrtiko	–
12			Vilana	–
13			Vidiano	–
14			Dafni	Dafnia
15			Thrapsathiri	–
16			Moschato Aspro	Moschato Spinas; Moschato Mazas
17			Plito	–
18			Chardonnay	–
19			Grenache Blanc	–
20			Sauvignon Blanc	–
1	**Permitted**	Red	Kotsifoliatiko	–
2			Romeiko	–
3			Tsardana	–
4			Fokiano	–
5			Alicante Bouschet	–
6			Mourvedre	–
7			Merlot	–
8		White	Valaitis	–
9			Dermatas	–
10			Razaki	Kerino
11			Soultanina	–
12			Malvasia di Candia Aromatica	–
13			Maccabeu	–
14			Sylvaner	–
15			Ugni Blanc	Trebbiano
16			Viognier	–
1	**Temporarily permitted**	Red	Montepulciano	–
2			Petit Verdot	–
3		White	Roussanne	–

Table S3 . Classical oenological parameters of Cretan red wines by variety (mean ± SD).

Parameter	Kotsifali (n=6)	Liatiko (n=12)	Mandilaria (n=4)	Romeiko (n=3)	Welch p	η^2
Phenolic Content Index (PCI)	42.0 ± 9.9^{ab}	42.2 ± 9.6^{ab}	84.7 ± 19.7^{a}	33.7 ± 2.1^{b}	0.0050	0.705
Tannins (g/L)	3.10 ± 1.30^{bc}	3.05 ± 0.81^{b}	7.36 ± 1.46^{a}	1.83 ± 0.15^{c}	<0.001	0.755
Color Intensity (I)	6.6 ± 2.6b	5.4 ± 2.0b	12.3 ± 2.5a	3.4 ± 2.7ab	0.0500	0.616
Color Hue (T)	1.10 ± 0.13^{a}	1.15 ± 0.12^{a}	0.79 ± 0.07^{b}	1.37 ± 0.52^{ab}	0.0150	0.510
Total Phenolic Content (Folin–Ciocalteu, $mg \cdot L^{-1}$GAE)	2410 ± 790	2610 ± 700	4310 ± 1620	1350 ± 760	0.0980	0.483
Free SO_2 ($mg \cdot L^{-1}$)	49.1 ± 8.5	43.4 ± 11.4	49.6 ± 8.5	50.2 ± 11.2	0.6720	0.096
Total SO_2 ($mg \cdot L^{-1}$)	142 ± 25	133 ± 30	138 ± 24	169 ± 45	0.7090	0.144
Bound (Combined) SO_2 ($mg \cdot L^{-1}$)	93 ± 20	75 ± 47	88 ± 17	118 ± 37	0.4930	0.140
Titratable Acidity ($g \cdot L^{-1}$)	5.07 ± 0.44	4.43 ± 0.59	4.95 ± 1.07	4.70 ± 0.35	0.2140	0.187
pH	3.45 ± 0.13	3.54 ± 0.21	3.42 ± 0.18	3.50 ± 0.06	0.6340	0.086
Reducing sugars ($g \cdot L^{-1}$)	1.03 ± 0.76	1.33 ± 0.92	1.18 ± 0.73	1.14 ± 0.71	0.9220	0.026

Note: Values are mean ± SD. Welch's one-way ANOVA was applied across varieties; when significant, Games–Howell post-hoc testing was used. Different superscript letters within the same row indicate significant differences among varieties ($p < 0.05$).

Table S4. Classical oenological parameters of Cretan white wines by variety (mean ± SD).

**Parameter**	**Assyrtiko (n=5)**	**Dafni (n=2)**	**Malvazia (n=2)**	**Melissaki (n=2)**	**Moschato Spinas (n=4)**	**Plito (n=3)**	**Romeiko (n=3)**	**Vidiano (n=17)**	**Vilana (n=4)**	**Welch p**	**η^2**
Total Phenolic Content (Folin–Ciocalteu, $mg \cdot L^{-1}$ GAE)	540 ± 180	310 ± 60	330 ± 120	1180 ± 270	350 ± 40	460 ± 30	400 ± 10	480 ± 140	470 ± 70	0.068	0.684
Free SO_2 ($mg \cdot L^{-1}$)	35.5 ± 27.6	38.4 ± 18.1	64.1 ± 18.1	20.0 ± 7.9	85.4 ± 14.8	59.8 ± 29.6	56.6 ± 18.2	36.0 ± 15.7	28.4 ± 15.6	0.063	0.501
Total SO_2 ($mg \cdot L^{-1}$)	97 ± 37^{bc}	74 ± 3^{b}	179 ± 36^{ab}	65 ± 17^{ab}	188 ± 15^{a}	137 ± 39^{ab}	163 ± 14^{ac}	139 ± 29^{ac}	105 ± 25^{bc}	<0.001	0.641
Bound (Combined) SO_2 ($mg \cdot L^{-1}$)	61 ± 31^{a}	36 ± 15^{a}	115 ± 18^{a}	45 ± 9^{a}	77 ± 51^{a}	77 ± 26^{a}	107 ± 7^{a}	85 ± 45^{a}	77 ± 20^{a}	0.01	0.218
Titratable Acidity ($g \cdot L^{-1}$)	5.77 ± 0.72	5.29 ± 0.16	5.33 ± 0.53	5.03 ± 0.11	6.34 ± 0.65	5.50 ± 0.37	5.51 ± 0.34	5.40 ± 0.64	5.39 ± 1.03	0.175	0.222
pH	2.95 ± 0.11	3.30 ± 0.16	3.14 ± 0.08	3.46 ± 0.11	3.18 ± 0.12	3.16 ± 0.13	3.19 ± 0.15	3.31 ± 0.20	3.31 ± 0.17	0.063	0.427
Absorbance at 420 nm (A_{420})	0.105 ± 0.052^{bc}	0.116 ± 0.045^{abc}	0.105 ± 0.014^{c}	0.462 ± 0.025^{s}	0.064 ± 0.032^{c}	0.119 ± 0.016^{bc}	0.116 ± 0.029^{bc}	0.178 ± 0.059^{b}	0.133 ± 0.040^{bc}	<0.001	0.768
Reducing sugars ($g \cdot L^{-1}$)	2.99 ± 3.21	1.23 ± 0.26	2.77 ± 2.41	0.54 ± 0.48	2.57 ± 1.72	1.88 ± 1.89	1.38 ± 0.36	2.43 ± 1.35	1.07 ± 0.47	0.252	0.176

Note: Values are mean ± SD. Welch's one-way ANOVA was applied across varieties; when significant, Games–Howell post-hoc testing was used. Different superscript letters within the same row indicate significant differences among varieties ($p < 0.05$).

Supplementary Method S1. PCA diagnostics and cross-validation (red wines)

For the PCA model built on the phenolic matrix of red wines, sampling adequacy and sphericity were evaluated using the Kaiser–Meyer–Olkin (KMO) measure and Bartlett's test. The overall KMO was 0.12. Bartlett's test indicated that the correlation matrix significantly differed from an identity matrix ($\chi^2 = 586.31$, df = 253, $p = 1.67 \times 10^{-28}$).

Table S5. Variance explained by PCA components (red wines).

PC	Var. explained (%)	Cumulative (%)
1	30.01	30.01
2	19.36	49.37
3	18.52	67.89
4	9.70	77.58
5	3.66	81.25
6	3.52	84.77
7	3.00	87.77
8	2.66	90.43
9	2.18	92.61
10	1.98	94.59

Table S6. Variance explained by PCA components (red wines).

PCs	Q^2
1	0.169
2	0.265
3	0.519
4	0.648
5	0.656
6	0.676
7	0.700
8	0.736
9	0.770
10	0.793

Supplementary Method S2. PLS-DA modelling details

PLS-DA was performed in mixOmics using *plsda()*. The optimal number of latent components was selected by repeated M-fold cross-validation implemented with *perf()* (Mfold; 5 folds, 50 repeats; dist = "max.dist"; set.seed(123)), by minimising the balanced error rate (BER) across components. Variable importance was summarised using VIP scores computed with *vip()*; VIP rankings correspond to the selected component of the final fitted model (vip[, best_ncomp]). For descriptive purposes, a confusion matrix was additionally generated from in-sample class predictions using predict(..., dist = "max.dist").

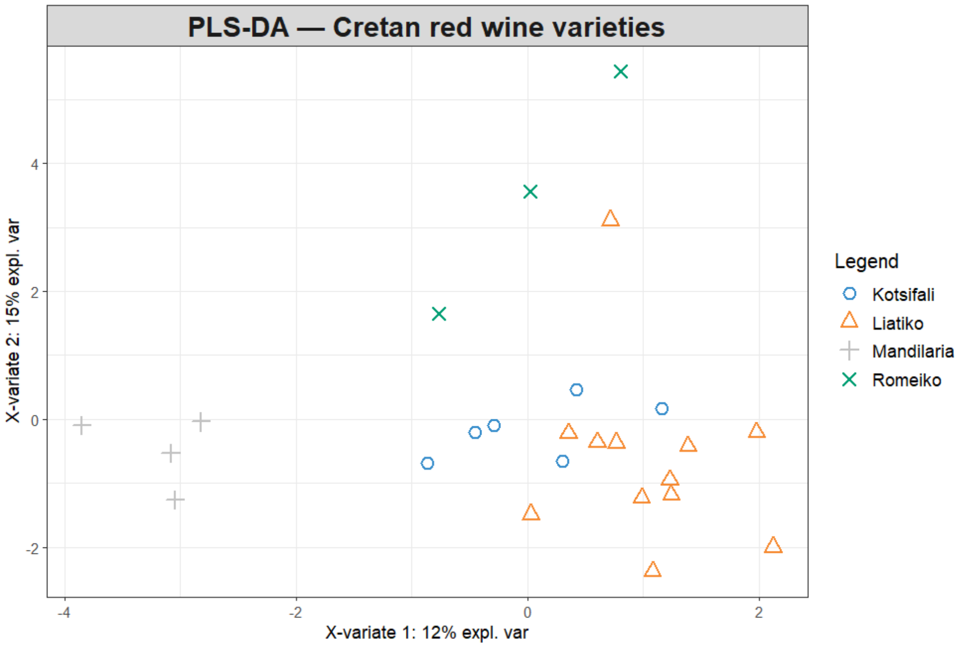

(a)

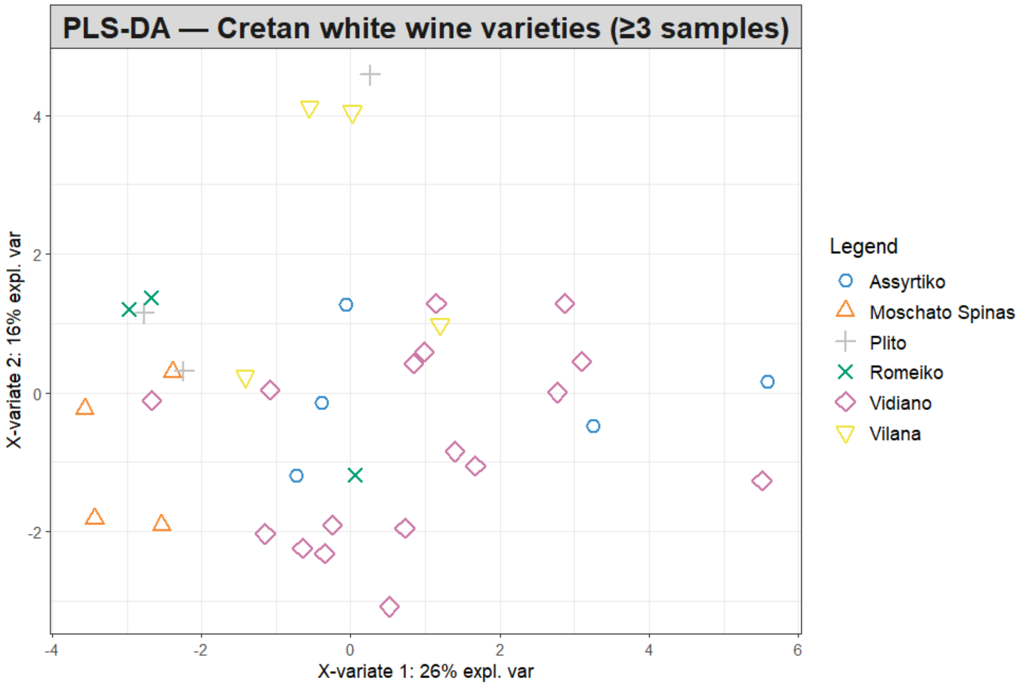

(b)

Figure S1. PLS-DA score plots based on the targeted phenolic composition (LC–QTOF-MS) of Cretan wines. (a) Cretan red wine varieties (Kotsifali, Liatiko, Mandilaria, Romeiko); (b) Cretan white wine varieties including only classes with ≥3 samples (Assyrtiko, Moschato Spinas, Plito, Romeiko, Vidiano, Vilana). Each

point represents an individual wine sample, coloured/symbol-coded by variety; axes correspond to the first two latent variables (X-variates), with the percentage of explained X-variance indicated on the plot.

Table S7. Cross-validated BER by component (red wines).

Components	BER
1	0.6308
2	0.5792
3	0.4400

Table S8. Confusion matrix for the final red-wine PLS-DA model (in-sample prediction; descriptive).

True \ Pred	Kotsifali	Liatiko	Mandilaria	Romeiko
Kotsifali	4	2	0	0
Liatiko	0	11	0	1
Mandilaria	0	0	4	0
Romeiko	0	0	0	3

Table S9. Top VIP-ranked phenolic predictors in the final red-wine PLS-DA model (selected component).

Rank	Feature	VIP
1	Protocatechuic acid ethyl ester	1.398
2	Tyrosol	1.348
3	Vanillin	1.338
4	Kaempferol	1.297
5	Quercetin	1.276
6	Caffeic acid	1.275
7	Hydroxybenzaldehyde	1.247
8	Taxifolin	1.130
9	Gentisic acid	1.123
10	Catechin	1.115

Table S10. Concentrations ($mg \cdot L^{-1}$) of target compounds in Cretan white wines and univariate statistics (Welch's ANOVA and η^2) calculated after excluding Melissaki.

**Flavonoid / Non-flavonoid**	**Phenolic category**	**Compound**	**Vidiano (n=17)**	**Assyrtiko (n=5)**	**Vilana (n=4)**	**Moschato Spinas (n=4)**	**Plito (n=3)**	**Romeiko (n=3)**	**Dafni (n=2)**	**Malvazia (n=2)**	**Welch p**	**η^2**
Flavonoid	Flavan-3-ol (flavanol)	Catechin	3.748 ± 2.352^{a}	6.078 ± 4.340^{ab}	4.005 ± 1.993^{ab}	0.744 ± 0.572^{b}	3.494 ± 3.476^{ab}	1.499 ± 0.905^{ab}	3.631 ± 0.061^{a}	1.000 ± 0.639^{ab}	0.001	0.307
Flavonoid	Flavan-3-ol (flavanol)	Epicatechin	1.015 ± 0.809^{ab}	1.675 ± 1.405^{a}	1.316 ± 0.718^{a}	0.303 ± 0.255^{b}	1.145 ± 1.088^{ab}	0.264 ± 0.227^{b}	0.854 ± 0.089^{ab}	0.323 ± 0.098^{b}	0.019	0.236
Flavonoid	Flavanone	Eriodictyol	<LOQ	<LOQ	<LOQ	<LOQ	0.123 ± 0.130	<LOQ	<LOQ	<LOQ	.	0.262
Flavonoid	Flavanone	Hesperetin	<LOQ	<LOQ	N.D.	N.D.	N.D.	N.D.	N.D.	N.D.	.	.
Flavonoid	Flavanone	Naringenin	19.333 ± 18.231	21.058 ± 30.591	8.160 ± 7.628	3.531 ± 2.839	34.093 ± 38.419	6.747 ± 2.846	7.524 ± 3.153	10.338 ± 5.436	0.391	0.166
Flavonoid	Flavanone	Pinocembrin	<LOQ	<LOQ	<LOQ	<LOQ	0.130 ± 0.221	<LOQ	<LOQ	<LOQ	.	0.269
Flavonoid	Flavanone	Sakuranetin	0.800 ± 0.405	1.465 (n=1)	0.404 (n=1)	N.D.	11.447 (n=1)	N.D.	N.D.	N.D.	.	0.998
Flavonoid	Flavanonol	Pinobanksin	19.588 ± 18.106	22.243 ± 30.594	8.611 ± 7.911	3.960 ± 2.513	24.155 ± 32.937	7.039 ± 2.921	8.180 ± 3.840	10.983 ± 5.781	0.301	0.137
Flavonoid	Flavanonol	Taxifolin	<LOQ	0.122 ± 0.066	0.174 ± 0.094	<LOQ	0.129 ± 0.087	0.128 ± 0.097	<LOQ	<LOQ	0.594	0.213
Flavonoid	Flavone	Chrysin	<LOQ	<LOQ	<LOQ	N.D.	<LOQ	N.D.	N.D.	N.D.	.	.
Flavonoid	Flavone	Luteolin	<LOQ	<LOQ	<LOQ	N.D.	N.D.	N.D.	<LOQ	N.D.	.	.
Flavonoid	Flavonol	Galangin	0.005 (n=1)	0.023 ± 0.007	N.D.	N.D.	N.D.	N.D.	0.005 (n=1)	N.D.	.	0.789
Flavonoid	Flavonol	Kaempferol	<LOQ	<LOQ	<LOQ	<LOQ	<LOQ	<LOQ	<LOQ	<LOQ	.	.
Flavonoid	Flavonol	Quercetin	0.158 ± 0.153	0.718 ± 0.699	<LOQ	<LOQ	<LOQ	<LOQ	<LOQ	<LOQ	0.528	0.415
Non-flavonoid	Benzoic acid	3-hydroxybenzoic acid	0.288 ± 0.183	<LOQ	0.507 (n=1)	N.D.	<LOQ	<LOQ	0.315 (n=1)	N.D.	.	0.462

Non-flavonoid	Benzoic acid	4-hydroxybenzoic acid	<LOQ	N.D.	<LOQ	N.D.	<LOQ	N.D.	N.D.	<LOQ	.	.
Non-flavonoid	Benzoic acid	Gallic acid	3.638 ± 4.601	6.494 ± 6.681	1.089 ± 1.243	0.121 ± 0.088	0.249 ± 0.168	3.215 (n=1)	1.136 ± 0.158	1.192 (n=1)	.	0.208
Non-flavonoid	Benzoic acid	Gentisic acid	0.172 ± 0.171	0.213 ± 0.221	0.197 ± 0.211	<LOQ	<LOQ	<LOQ	<LOQ	<LOQ	0.245	0.154
Non-flavonoid	Benzoic acid	Protocatechuic acid	0.100 ± 0.045	0.116 ± 0.038	0.219 ± 0.194	<LOQ	0.114 ± 0.040	0.126 ± 0.042	0.133 ± 0.041	0.105 (n=1)	.	0.293
Non-flavonoid	Benzoic acid	Salicylic acid	0.323 ± 0.427	0.299 ± 0.362	0.290 ± 0.201	0.304 ± 0.157	0.336 ± 0.098	0.220 ± 0.094	0.193 ± 0.156	0.645 ± 0.430	0.870	0.067
Non-flavonoid	Benzoic acid derivative	Ethyl gallate	2.534 ± 2.986	3.330 ± 2.570	2.331 ± 2.327	0.173 ± 0.107	1.152 ± 1.138	0.761 ± 0.597	2.287 ± 1.312	1.752 ± 2.040	0.125	0.15
Non-flavonoid	Benzoic acid derivative	Protocatechuic acid ethyl ester	<LOQ	<LOQ	0.170 ± 0.174	<LOQ	<LOQ	0.109 ± 0.014	0.140 ± 0.032	<LOQ	.	.
Non-flavonoid	Benzoic aldehyde	Hydroxybenzaldehyde	<LOQ	<LOQ	0.102 ± 0.125	<LOQ	<LOQ	0.027 ± 0.030	<LOQ	<LOQ	.	.
Non-flavonoid	Benzoic aldehyde	Syringaldehyde	0.210 ± 0.055	0.479 (n=1)	N.D.	N.D.	N.D.	0.458 (n=1)	0.379 ± 0.088	N.D.	.	0.865
Non-flavonoid	Benzoic aldehyde	Vanillin	0.302 ± 0.107	0.305 ± 0.095	0.150 ± 0.123	0.173 ± 0.090	0.262 ± 0.034	0.252 ± 0.026	0.104 (n=1)	0.162 ± 0.026	.	0.356
Non-flavonoid	Hydroxycinnamate ester	Ethyl caffeate	22.575 ± 31.160^{b}	28.017 ± 42.945^{ab}	44.940 ± 42.398^{ab}	43.580 ± 47.229^{ab}	69.967 ± 12.451^{a}	21.337 ± 19.319^{ab}	3.018 ± 3.718^{b}	45.987 ± 43.962^{ab}	0.006	0.2
Non-flavonoid	Hydroxycinnamic acid	Caffeic acid	5.998 ± 3.624	8.238 ± 6.754	9.374 ± 2.257	3.072 ± 2.628	6.405 ± 0.712	2.562 ± 0.816	0.809 ± 0.385	1.731 (n=1)	.	0.334
Non-flavonoid	Hydroxycinnamic acid	Cinnamic acid	0.495 ± 0.184	0.513 ± 0.170	0.246 ± 0.142	0.517 ± 0.384	0.539 ± 0.199	0.545 ± 0.080	1.199 (n=1)	0.468 ± 0.594	.	0.387
Non-flavonoid	Hydroxycinnamic acid	Ferulic acid	N.D.	<LOQ	N.D.	N.D.	N.D.	N.D.	N.D.	<LOQ	.	0.608
Non-flavonoid	Hydroxycinnamic acid	p-coumaric acid	0.811 ± 0.830	1.345 ± 1.203	0.630 ± 0.498	0.310 ± 0.258	0.456 ± 0.140	0.187 ± 0.235	0.108 ± 0.133	0.164 ± 0.115	0.153	0.222

Non-flavonoid	Phenolic acid derivative	Homovanillic acid	1.129 ± 0.936	1.178 ± 1.107	2.071 ± 0.235	1.608 ± 0.071	2.979 ± 0.766	1.377 ± 1.163	3.838 (n=1)	1.477 ± 0.040	.	0.496
Non-flavonoid	Phenylethanol derivative	Hydroxytyrosol	0.716 ± 0.729[b]	1.114 ± 0.923ab	0.882 ± 0.763[ab]	1.804 ± 1.065[ab]	2.822 ± 0.641[ab]	2.103 ± 1.642[ab]	0.296 ± 0.163[b]	2.257 ± 0.206[a]	<0.001	0.462
Non-flavonoid	Phenylethanol derivative	Tyrosol	16.975 ± 6.839	11.319 ± 4.451	13.004 ± 6.301	12.345 ± 6.273	9.938 ± 0.564	14.615 ± 7.173	13.474 ± 6.770	17.792 ± 4.916	0.181	0.178
Non-flavonoid	Simple phenol	Catechol	<LOQ	<LOQ	<LOQ	<LOQ	<LOQ	<LOQ	<LOQ	<LOQ	.	0.378
Non-flavonoid	Stilbene	Resveratrol	0.104 ± 0.079	0.071 ± 0.029	0.226 ± 0.146	<LOQ	<LOQ	0.124 ± 0.010	0.322 ± 0.240	<LOQ	.	0.450

Note: Values are mean ± SD. Welch's one-way ANOVA was applied across varieties; when significant, Games–Howell post-hoc testing was used. Different superscript letters within the same row indicate significant differences among varieties ($p < 0.05$).

Supplementary Method S3. PCA and MFA models for Cretan white wines

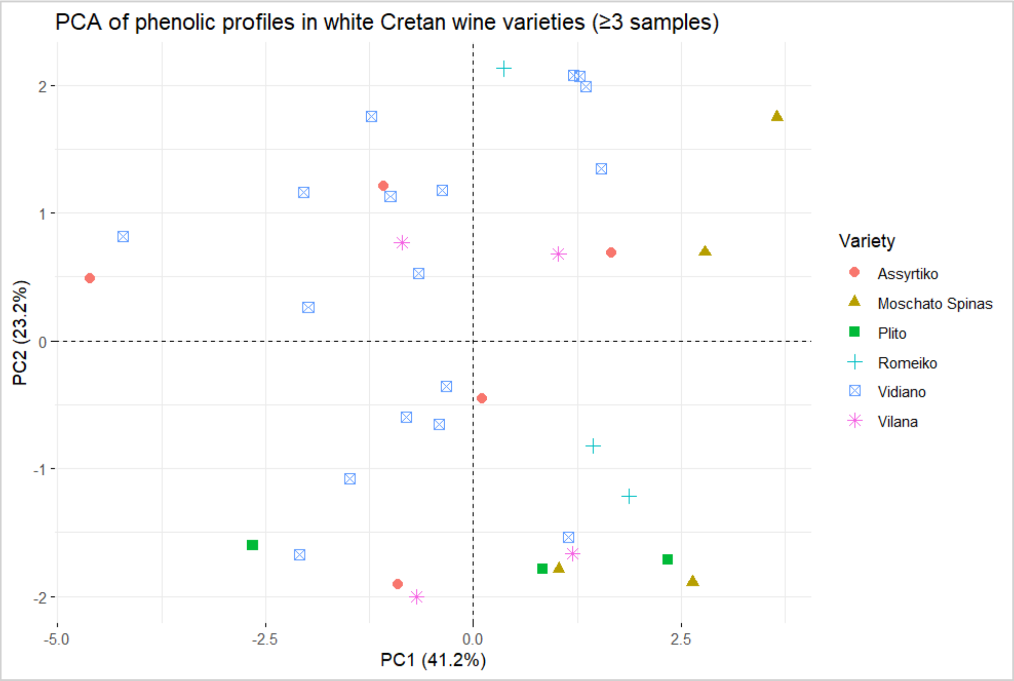

(**a**)

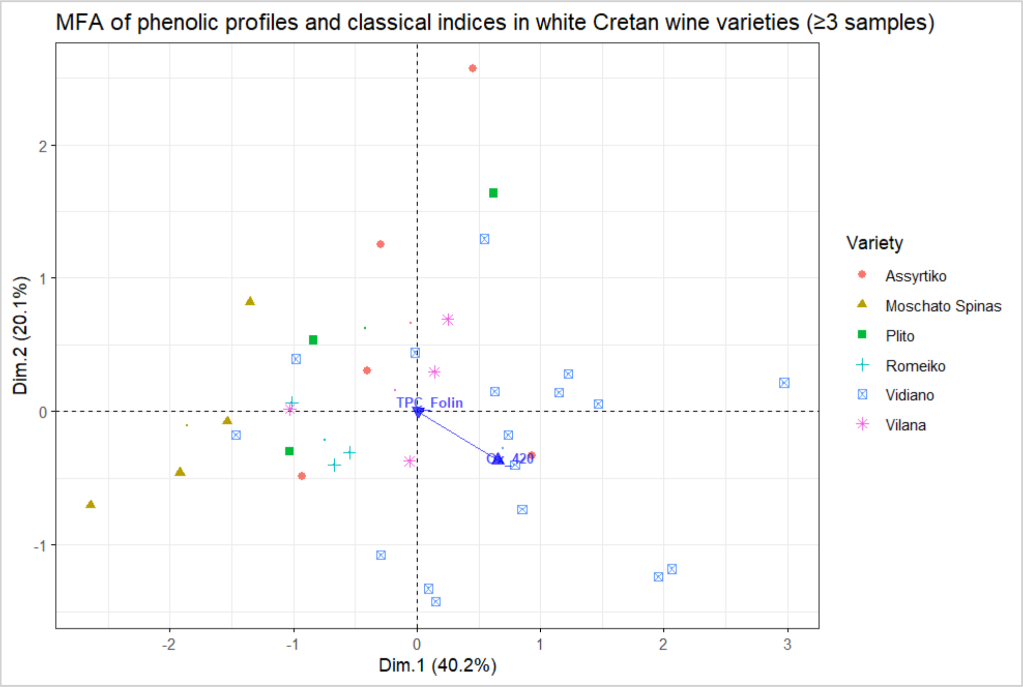

(**b**)

Figure S2. Multivariate analysis of Cretan white wines by variety: (a) Principal component analysis (PCA) score plot based on phenolic composition for varieties with ≥4 wines, with 95% confidence ellipses for Assyrtiko, Moschato Spinas, Vidiano and Vilana; (b) Multiple factor analysis (MFA) compromise map integrating the targeted phenolic block with classical indices (total phenolics by Folin–Ciocalteu, TPC_Folin,

and absorbance at 420 nm, A_{420}/Ox_420), with the corresponding loading vectors projected onto the sample space.

Supplementary Method S4. PCA diagnostics and cross-validation (white wines, varieties with ≥3 wines)

For the PCA model built on the phenolic matrix of white wines (restricted to grape varieties represented by ≥3 wines), sampling adequacy and sphericity were assessed using the Kaiser–Meyer–Olkin (KMO) measure and Bartlett's test. The overall KMO was 0.51. Bartlett's test indicated that the correlation matrix significantly differed from an identity matrix (χ^2= 749.89, df = 253, p = 1.12×10^{-50}).

Table S11. Variance explained by PCA components (white wines; ≥3 wines/variety).

PC	Var. explained (%)	Cumulative (%)
1	41.18	41.18
2	23.21	64.39
3	13.07	77.46
4	8.64	86.10
5	4.06	90.15
6	2.17	92.32
7	1.77	94.09
8	1.22	95.31
9	0.92	96.22
10	0.80	97.02

Table S12. Cross-validated Q^2 (reconstruction) by number of PCs (white wines; ≥3 wines/variety).

PCs	Q^2
1	0.340
2	0.599
3	0.728
4	0.807
5	0.854
6	0.866
7	0.894
8	0.906
9	0.913
10	0.925

Table S13. Cross-validated BER by component (white wines).

Components	BER
1	0.7854
2	0.7826
3	0.7466
4	0.7153
5	0.7031

Table S14. Confusion matrix for the final white-wine PLS-DA model (in-sample prediction; descriptive).

True \ Pred	Assyrtiko	Moschato Spinas	Plito	Vidiano	Vilana
Assyrtiko	2	0	0	3	0
Moschato Spinas	0	3	0	1	0
Plito	0	1	2	0	0
Romeiko	0	0	2	1	0
Vidiano	0	1	0	16	0
Vilana	0	0	0	0	4

Table S15. Top VIP-ranked phenolic predictors in the final white-wine PLS-DA model (selected component).

Rank	Feature	VIP
1	Quercetin	1.594
2	Resveratrol	1.243
3	Vanillin	1.203
4	Hydroxytyrosol	1.149
5	Eriodictyol	1.108
6	Protocatechuic acid	1.103
7	Protocatechuic acid ethyl ester	1.070
8	Tyrosol	1.061
9	Naringenin	1.027
10	Pinocembrin	1.027

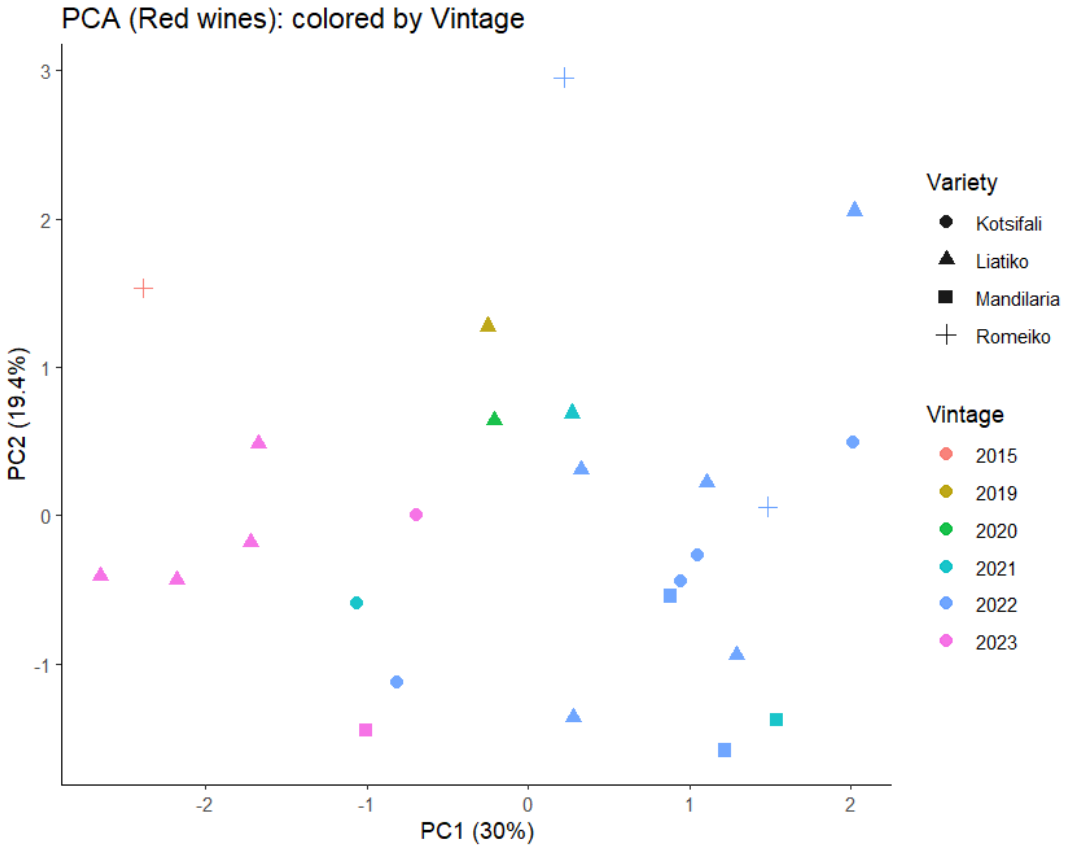
PCA (Red wines): colored by Vintage
PC2 (19.4%)
PC1 (30%)
3
2
1
0
-1
-2
-1
0
1
2
Variety
Kotsifali
Liatiko
Mandilaria
Romeiko
Vintage
2015
2019
2020
2021
2022
2023
(a)

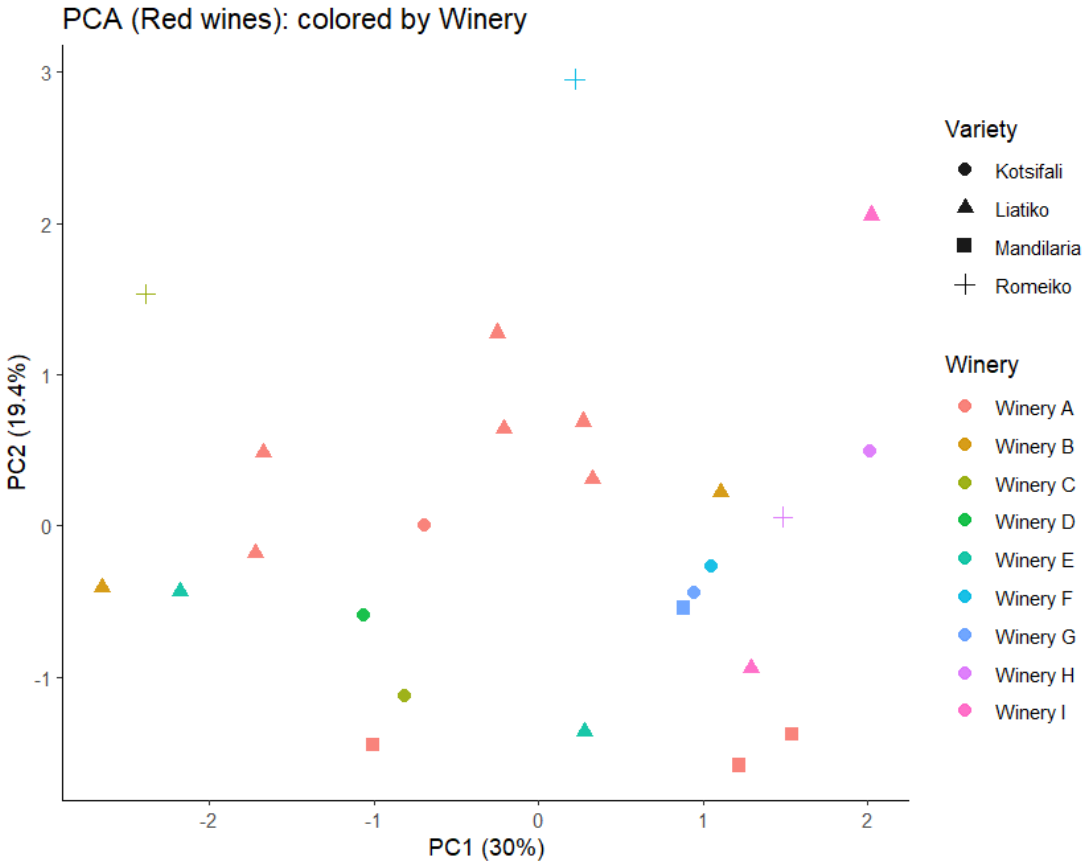
PCA (Red wines): colored by Winery
PC2 (19.4%)
PC1 (30%)
3
2
1
0
-1
-2
-1
0
1
2
Variety
Kotsifali
Liatiko
Mandilaria
Romeiko
Winery
Winery A
Winery B
Winery C
Winery D
Winery E
Winery F
Winery G
Winery H
Winery I
(b)

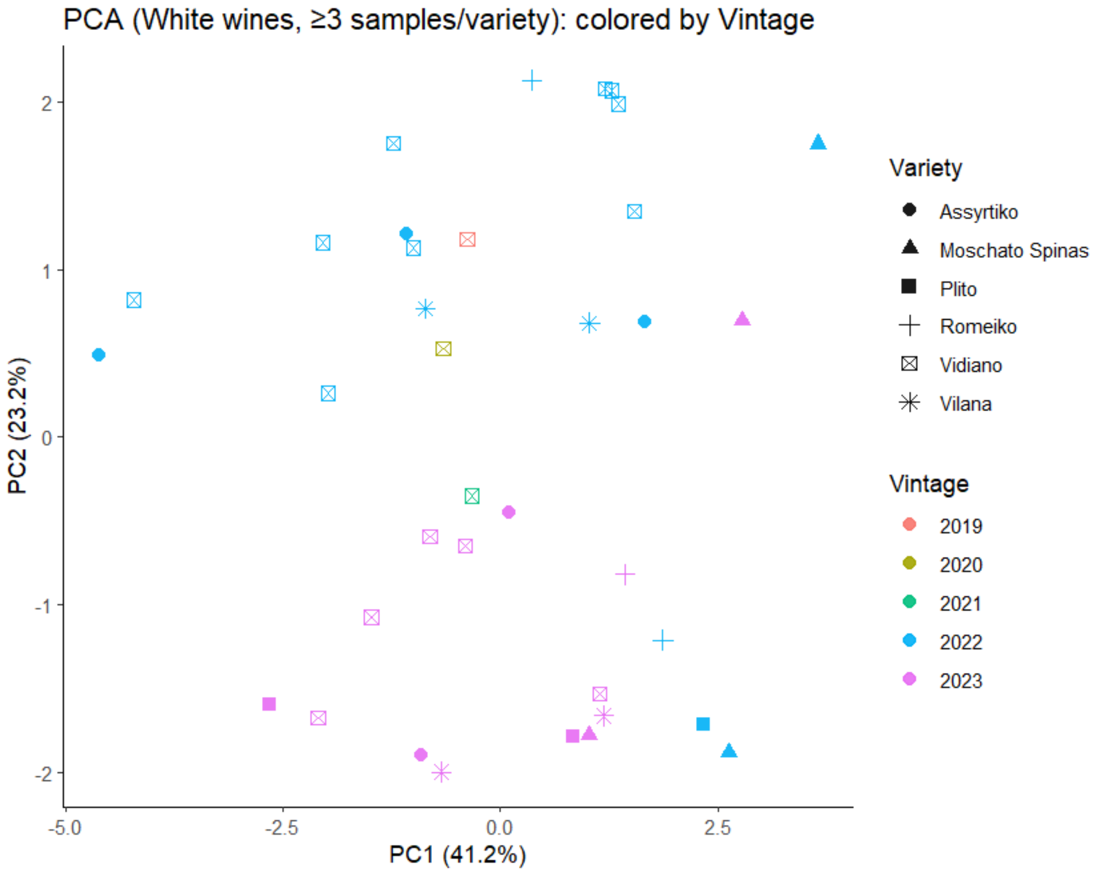
PCA (White wines, ≥3 samples/variety): colored by Vintage
PC2 (23.2%)
PC1 (41.2%)
2
1
0
-1
-2
-5.0
-2.5
0.0
2.5
Variety
Assyrtiko
Moschato Spinas
Plito
Romeiko
Vidiano
Vilana
Vintage
2019
2020
2021
2022
2023

(c)

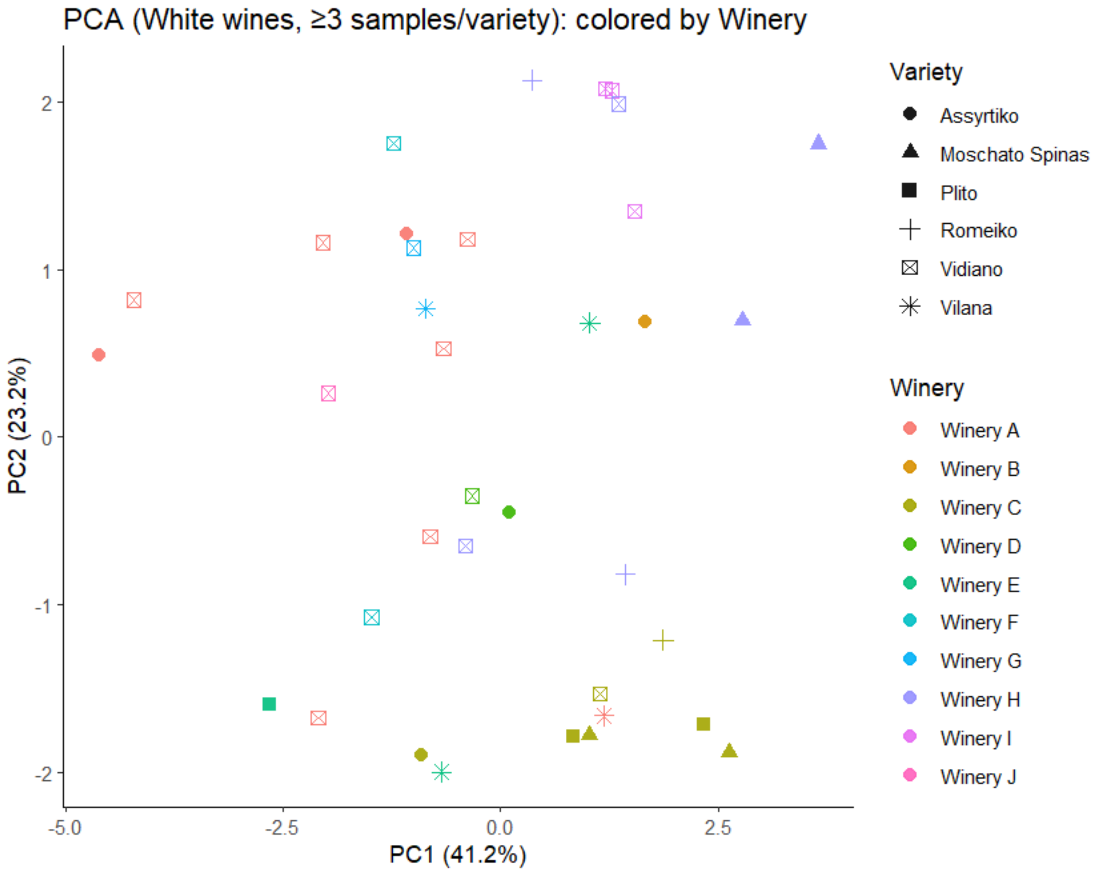

(d)

Figure S3. PCA sensitivity plots colored by vintage year and winery code. (a,b) Red wines; (c,d) white wines (varieties with ≥3 samples per variety). Colors indicate vintage year (a,c) or winery code (b,d), while shapes indicate variety. The variance explained by PC1 and PC2 is reported on the axes.

Table S16. Wine samples analyzed in the study: variety, color, winery code, vintage year, region, n (number of wine samples) and sample ID.

Variety	Wine color	Winery code	Vintage year	Region	n	Sample ID(s)
Assyrtiko	White	Winery A	2022	Heraklion	2	assyrt_1<br>assyrt_2
Assyrtiko	White	Winery B	2022	Heraklion	1	assyrt_3
Assyrtiko	White	Winery C	2023	Chania	1	assyrt_4
Assyrtiko	White	Winery D	2023	Heraklion	1	assyrt_5
Dafni	White	Winery E	2022	Heraklion	1	dafni_1
Dafni	White	Winery E	2023	Heraklion	1	dafni_2
Kotsifali	Red	Winery D	2021	Heraklion	1	kotsif_1
Kotsifali	Red	Winery F	2022	Chania	1	kotsif_2
Kotsifali	Red	Winery G	2022	Heraklion	1	kotsif_3
Kotsifali	Red	Winery H	2022	Chania	1	kotsif_4
Kotsifali	Red	Winery C	2022	Chania	1	kotsif_5
Kotsifali	Red	Winery A	2023	Chania	1	kotsif_6
Liatiko	Red	Winery A	2019	Heraklion	1	liat_1
Liatiko	Red	Winery A	2020	Heraklion	1	liat_2
Liatiko	Red	Winery A	2021	Heraklion	1	liat_3
Liatiko	Red	Winery A	2022	Heraklion	1	liat_4
Liatiko	Red	Winery B	2022	Heraklion	1	liat_5
Liatiko	Red	Winery E	2022	Heraklion	1	liat_6
Liatiko	Red	Winery I	2022	Rethymno	2	liat_7<br>liat_8
Liatiko	Red	Winery A	2023	Heraklion	2	liat_9<br>liat_10
Liatiko	Red	Winery B	2023	Heraklion	1	liat_11
Liatiko	Red	Winery E	2023	Heraklion	1	liat_12
Malvazia	White	Winery C	2022	Chania	1	malvazia_1
Malvazia	White	Winery C	2023	Chania	1	malvazia_2
Mandilaria	Red	Winery A	2021	Heraklion	1	mand_1
Mandilaria	Red	Winery A	2022	Heraklion	1	mand_2
Mandilaria	Red	Winery G	2022	Heraklion	1	mand_3
Mandilaria	Red	Winery A	2023	Heraklion	1	mand_4
Melissaki	White	Winery E	2022	Heraklion	1	meliss_1
Melissaki	White	Winery E	2023	Heraklion	1	meliss_2
Moschato Spinas	White	Winery C	2022	Chania	1	mosx.sp_1
Moschato Spinas	White	Winery H	2022	Chania	1	mosxat_1

Moschato Spinas	White	Winery C	2023	Chania	1	mosx.sp_3
Moschato Spinas	White	Winery H	2023	Chania	1	mosx.sp_2
Plito	White	Winery C	2022	Chania	1	plito_1
Plito	White	Winery C	2023	Chania	1	plito_3
Plito	White	Winery E	2023	Heraklion	1	plito_2
Romeiko	Red	Winery C	2015	Chania	1	romeik_6
Romeiko	Red	Winery F	2022	Chania	1	romeik_1
Romeiko	Red	Winery H	2022	Chania	1	romeik_2
Romeiko	White	Winery C	2022	Chania	1	romeik_4
Romeiko	White	Winery H	2022	Chania	1	romeik_3
Romeiko	White	Winery H	2023	Chania	1	romeik_5
Vidiano	White	Winery A	2019	Heraklion	1	vidian_1
Vidiano	White	Winery A	2020	Heraklion	1	vidian_2
Vidiano	White	Winery D	2021	Heraklion	1	vidian_3
Vidiano	White	Winery A	2022	Heraklion	2	vidian_4
						vidian_5
Vidiano	White	Winery F	2022	Chania	1	vidian_7
Vidiano	White	Winery G	2022	Heraklion	1	vidian_8
Vidiano	White	Winery H	2022	Chania	1	vidian_12
Vidiano	White	Winery I	2022	Rethymno	3	vidian_9
						vidian_10
						vidian_11
Vidiano	White	Winery J	2022	Heraklion	1	vidian_6
Vidiano	White	Winery A	2023	Heraklion	2	vidian_13
						vidian_14
Vidiano	White	Winery C	2023	Chania	1	vidian_17
Vidiano	White	Winery F	2023	Chania	1	vidian_15
Vidiano	White	Winery H	2023	Chania	1	vidian_16
Vilana	White	Winery E	2022	Heraklion	1	vilan_2
Vilana	White	Winery G	2022	Heraklion	1	vilan_1
Vilana	White	Winery A	2023	Heraklion	1	vilan_3
Vilana	White	Winery E	2023	Heraklion	1	vilan_4

Supplementary Methods S5. Classical oenological analysis and spectrophotometric indices

S5.1 Materials for Classical Analysis

All reagents and solvents used in this study were of analytical grade. Sulfuric acid (H_2SO_4, 96%) and hydrochloric acid (HCl, 37%) were obtained from Panreac Química S.A. (Barcelona, Spain). Starch (from potatoes) was supplied by Lach-Ner, s.r.o. (Neratovice, Czech Republic), iodine solution (0.05 mol/L) was purchased from Chem-Lab NV (Zedelgem, Belgium), potassium hydroxide (KOH, pellets) and sodium hydroxide (NaOH, >99%) were obtained from Merck KGaA (Darmstadt, Germany). Phenolphthalein was supplied by Carlo Erba Reagents S.A.S. (Val de Reuil Cedex, France), absolute ethanol was purchased from Fisher Chemical (Loughborough, UK), and the gallic acid standard (monohydrate) was obtained from Acros Organics (Geel, Belgium). Sodium carbonate (Na_2CO_3, anhydrous) was supplied by Chem-Lab NV (Zedelgem, Belgium), and the Folin-Ciocalteu reagent was purchased from VWR Chemicals BDH (Leuven, Belgium). The BioSystems D-Glucose/D-Fructose Kit (Cod. 12800) was supplied by BioSystems S.A. (Barcelona, Spain), and bromothymol blue was obtained from Thermo Scientific Chemicals (Waltham, MA, USA). Deionized water was used for all dilutions and sample preparations.

S5.2 Instrumentation

The instrumental analyses were conducted using a pH meter (model: HANNA edge HI2002), a spectrophotometer (UV-Vis, model: Shimadzu UV-1800 240V IVDD) for absorbance measurements at 280 nm, 340 nm, 420 nm, 520 nm, 550 nm, 620 nm, and 750 nm, and a centrifuge (model: REMI NEYA 8) operating at 6000 rpm for sample clarification. A thermostated water bath (model: Memmert WNB 45) was used for analyses requiring precise temperature control at 37°C and 100°C.

S5.3 Free and Total Sulfur Dioxide content

Sulfur dioxide content was determined according to the OIV-MA-AS323-04B method, which is based on titration of free sulfite with iodine using a starch solution as an indicator. For free sulfur dioxide, 25 mL of wine was transferred to a 250 mL flask, followed by the addition of 2.5 mL of H_2SO_4 and 0.5 mL of starch solution 10 g/L. Titration was performed with 0.02 M iodine solution until a blue color persisted for 10-15 seconds. Total sulfur dioxide was measured by transferring 25 mL of wine to a 500 mL flask, adding 12.5 mL of potassium hydroxide (KOH), and allowing the mixture to stand for 10 minutes. Afterward, 5 mL of H_2SO_4 and 0.5 mL of starch solution were added, and the titration was conducted with 0.02 M iodine solution until the color change was observed. The results were expressed as mg SO_2/L.

S5.4 Titratable Acidity

Titratable acidity was determined according to the OIV-MA-AS313-01 method. A 25 mL aliquot of wine was degassed to remove carbon dioxide and titrated with 0.1 N sodium hydroxide (NaOH) solution to a pH of 7.0. The endpoint was detected visually using bromothymol blue as the indicator. Results were expressed as g/L of tartaric acid equivalents.

S5.5 Active Acidity (pH)

Active acidity (pH) was determined according to the OIV-MA-AS313-15 method. Wine samples were measured at 20°C using a calibrated pH meter. The pH meter was calibrated using two standard buffer solutions bracketing the expected pH range of the samples. The electrode was immersed in the sample, and the pH was recorded directly from the meter display.

S5.6 Total tannins (boiling–acid method)

The total tannin content was determined using a spectrophotometric method based on the transformation of proanthocyanidins into anthocyanidins in an acidic medium at 100°C [42]. This method quantifies the total tannins that are converted into anthocyanidins, including those bound to other phenolic compounds. The results were corrected using specific coefficients (Glories, 1988) to account for potential procedural errors. For the analysis, a 1 mL aliquot of wine was diluted to 50 mL with deionized water in a volumetric flask. A 2 mL portion of the diluted wine was then transferred into a test tube, followed by the addition of 1 mL of deionized water and 3 mL of concentrated HCl (37%). One test tube was heated in a boiling water bath at 100°C for 45 minutes, while a second, unheated tube was kept at room temperature as a control. After cooling, 1 mL of 95% ethanol was added to both tubes to stabilize the color. The absorbance of each sample was measured at 550 nm using plastic cuvettes.

S5.7 Phenolic Content Index (PCI)

The phenolic content index (PCI) was determined based on the strong absorption of phenolic compounds in the ultraviolet region, with maximum absorption observed at 280 nm. This method is widely used due to its independence from pH variations and adherence to the Beer-Lambert law, making data collection straightforward. For the analysis, wine samples were centrifuged at 4000 rpm for 5 minutes. The supernatant was then diluted (1:100 or 1:150 depending on the intensity of the red color) in a 10 mL volumetric flask using deionized water. Absorbance was measured at 280 nm using a quartz cuvette with a 1 cm path length. The phenolic content index was calculated using the formula: PCI = OD × dilution factor.

S5.8 Folin-Ciocalteu Index

The total phenolic content (TPC) was determined using the Folin-Ciocalteu method according to the OIV-MA-AS2-10 protocol. This method is based on the oxidation of phenolic compounds by the Folin-Ciocalteu reagent, resulting in a blue complex with maximum absorbance at 750 nm, proportional to the phenolic content. For the analysis, a 1 mL aliquot of wine was placed in a 100 mL volumetric flask. Then, 50 mL of distilled water was added, followed by 5 mL of Folin-Ciocalteu reagent and 20 mL of 20% (m/v) sodium carbonate solution. The volume was brought to 100 mL with distilled water, and the solution was thoroughly mixed to ensure homogeneity. The solution was left to react at room temperature for 30 minutes. The absorbance was then measured at 750 nm using a spectrophotometer against a blank prepared with distilled water. The total phenolic content was calculated based on a gallic acid calibration curve, and results were expressed as $mg{\cdot}L^{-1}$of gallic acid equivalents (GAE). in $mg{\cdot}L^{-1}$.

S5.9 Reducing Sugars

Reducing sugars were determined using a commercial enzymatic kit (BioSystems, D-Glucose/D-Fructose, Cod. 12800) based on the hexokinase and phosphoglucose isomerase method. The method involves the enzymatic conversion of D-glucose and D-fructose to glucose-6-phosphate, followed by oxidation to gluconate-6-phosphate with the simultaneous reduction of $NADP^+$ to NADPH. The generated NADPH was quantified by measuring absorbance at 340 nm using a spectrophotometer.

For the analysis, reagents were prepared according to the manufacturer's instructions. The samples were pretreated by filtering and diluting as necessary to fall within the linear range of the assay. Measurements were performed at 37°C, and absorbance values were recorded after a 10-minute incubation period. The concentration of reducing sugars was calculated using a standard curve, and results were expressed as g/L of glucose equivalents.

S5.10 Color Characteristics (CI and Hue)

The chromatic properties of the wine samples were analyzed following the OIV-MA-AS2-11 methodology. After appropriate dilution to ensure measurements were within the linear range of the spectrophotometer, the absorbance of the diluted wine was measured at 420 nm, 520 nm, and 620 nm using a spectrophotometer with a 1 cm quartz cuvette.

Color intensity (CI) was calculated as the sum of the absorbance values at these wavelengths: $CI = A_{420} + A_{520} + A_{620}$.

Color hue (N) was calculated as the ratio of absorbance at 420 nm to 520 nm:

$H = A_{420} / A_{520}$.

Table S17. Target-screening database including precursor ion exact mass, retention time, and fragment ions

A/A	Compounds	Molecular Formula	[M-H]-	Rt (min)	Q1 m/z	Q1 formula	Q2 m/z	Q2 formula	Q3 m/z	Q3 formula
1	3-Hydroxybenzoic acid	$C_7H_6O_3$	138.0322	1.88	93.0346	C_6H_5O^1-	65.0397	C_5H_5^1-		
2	4-Hydroxybenzaldehyde	$C_7H_6O_2$	122.0373	4.77						
3	4-Hydroxybenzoic acid	$C_7H_6O_3$	138.0322	1.27	93.0346	C_6H_5O^1-	65.0397	C_5H_5^1-		
4	Apigenin	$C_{15}H_{10}O_5$	269.0455	7.90	117.0346	C_8H_5O^1−	151.0037	$C_7H_3O_4$^1−	149.0244	$C_8H_5O_3$^1−
5	Benzoic acid	$C_7H_6O_2$	121.0284	2.68	77.0397	C_6H_5^1-	93.0346	C_6H_5O^1-		
6	Caffeic acid	$C_9H_8O_4$	180.0428	2.53	135.0452	$C_8H_7O_2$^1-	134.0373	$C_8H_6O_2$^1-		
7	Catechin	$C_{15}H_{14}O_6$	290.0796	4.12	245.0819	$C_{14}H_{13}O_4$^1-	123.0452	$C_7H_7O_2$^1-	125.0244	$C_6H_5O_3$^1-
8	Catechol	$C_6H_6O_2$	110.0373	4.35	91.0189	C_6H_3O^1-	81.0346	C_5H_5O^1-		
9	Chlorogenic acid	$C_{16}H_{18}O_9$	354.0956	3.04	191.0561	$C_7H_{11}O_6$^1-	135.0452	$C_8H_7O_2$^1-	179.035	$C_9H_7O_4$^1-
10	Chrysin	$C_{15}H_{10}O_4$	253.0495	9.60	63.024	C_5H_3^1-	143.0502	$C_{10}H_7O$^1-	209.0608	$C_{14}H_9O_2$^1-
11	Cinnamic acid	$C_9H_8O_2$	148.053	3.97	103.0553	C_8H_7^1-	77.0397	C_6H_5^1-		
12	Diosmetin	$C_{16}H_{12}O_6$	299.0561	8.00	284.0326	$C_{15}H_8O_6$^1−	256.0377	$C_{14}H_8O_5$^1−	151.0037	$C_7H_3O_4$^1−
13	Epicatechin	$C_{15}H_{14}O_6$	290.0796	4.48	245.0819	$C_{14}H_{13}O_4$^1-	123.0452	$C_7H_7O_2$^1-	125.0244	$C_6H_5O_3$^1-
14	Eriodictyol	$C_{15}H_{12}O_6$	288.0639	5.96	135.0452	$C_8H_7O_2$^1-	151.0037	$C_7H_3O_4$^1-		
15	Ethyl caffeate	$C_{11}H_{12}O_4$	207.0652	7.14	135.0451	C8H7O2^1-	133.0296	C8H5O2^1-	161.0243	C9H5O3^1-
16	Ethyl gallate	$C_9H_{10}O_5$	198.0534	5.01	125.0244	$C_6H_5O_3$^1-	169.0142	$C_7H_5O_5$^1-		
17	Ferulic acid	$C_{10}H_{10}O_4$	193.0506	2.90	134.0373	C8H6O2^1-	178.0272	C9H6O4^1-	133.0295	C8H5O2^1
18	Galangin	$C_{15}H_{10}O_5$	269.0455	9.90	213.0557	$C_{13}H_9O_3$^1−	169.0659	$C_{12}H_9O$^1−	143.0502	$C_{10}H_7O$^1−
19	Gallic acid	$C_7H_6O_5$	171.0288	1.44	125.0244	$C_6H_5O_3$^1-	69.0346	C_4H_5O^1-	97.0295	$C_5H_5O_2$^1-
20	Gentisic acid	$C_7H_6O_4$	154.0272	2.28	108.0217	$C_6H_4O_2$^1-	109.0295	$C_6H_5O_2$^1-		
21	Hesperetin	$C_{16}H_{14}O_6$	302.0796	7.28	164.0115	$C_8H_4O_4$^1-	149.9947	$C_7H_3O_4$^1-	241.0495	$C_{14}H_{10}O_4$^1-

22	Homovanillic acid	$C_9H_{10}O_4$	182.0585	2.27	137.0608	$C_8H_9O_2$^1-	122.0373	$C_7H_6O_2$^1-		
23	Hydroxytyrosol	$C_8H_{10}O_3$	154.0635	3.83	123.0452	$C_7H_7O_2$^1-	95.0502	C_6H_7O^1-	122.0373	$C_7H_6O_2$^1-
24	Kaempferol	$C_{15}H_{10}O_6$	286.0483	8.00	229.0506	$C_{13}H_9O_4$^1-	185.0608	$C_{12}H_9O_2$^1-	159.0452	$C_{10}H_7O_2$^1-
25	Luteolin	$C_{15}H_{10}O_6$	286.0483	7.70	133.0295	$C_8H_5O_2$^1-	151.0037	$C_7H_3O_4$^1-	175.0401	$C_{10}H_7O_3$^1-
26	Myricetin	$C_{15}H_{10}O_8$	318.0381	6.11	151.0037	$C_7H_3O_4$^1-	178.9986	$C_8H_3O_5$^1-	137.0244	$C_7H_5O_3$^1-
27	Naringenin	$C_{15}H_{12}O_5$	271.0612	7.12	119.0502	$C_8H_7O^-$	151.0037	$C_7H_3O_4^-$	107.0138	$C_6H_3O_2^-$
28	p-Coumaric acid	$C_9H_8O_3$	164.0479	3.20	119.0502	C_8H_7O^1-	93.0346	C_6H_5O^1-		
29	Pinobanksin	$C_{15}H_{12}O_5$	271.0612	7.20	253.0495	$C_{15}H_9O_4$^1–	197.0597	$C_{13}H_9O_2$^1–	225.0546	$C_{14}H_9O_3$^1–
30	Pinocembrin	$C_{15}H_{12}O_4$	255.0663	9.08	151.0037	$C_7H_3O_4^-$	213.0557	$C_{13}H_9O_3^-$	107.0139	$C_6H_3O_2^-$
31	Protocatechuic acid	$C_7H_6O_4$	154.0272	1.65	109.0295	$C_6H_5O_2$^1-	108.0217	$C_6H_4O_2$^1-		
32	Protocatechuic acid Ethyl Ester	$C_9H_{10}O_4$	182.0585	6.03	109.0295	$C_6H_5O_2$^1-	153.0193	$C_7H_5O_4$^1-		
33	Quercetin	$C_{15}H_{10}O_7$	302.0432	7.14	151.0037	$C_7H_3O_4$^1-	178.9986	$C_8H_3O_5$^1-	121.0295	$C_7H_5O_2$^1-
34	Resveratrol	$C_{14}H_{12}O_3$	228.0792	5.81	143.0502	$C_{10}H_7O$^1-	185.0608	$C_{12}H_9O_2$^1-	159.0815	$C_{11}H_{11}O$^1-
35	Rosmarinic acid	C18H16O8	359.0772	4.30	161.0233	C9H5O3^1	197.0444	C9H9O5^1-	179.0338	C8H5O2^1
36	Rutin	$C_{27}H_{30}O_{16}$	610.1539	5.54	300.0276	$C_{15}H_8O_7$^1-	301.0354	$C_{15}H_9O_7$^1-	151.0037	$C_7H_3O_4$^1-
37	Sakuranetin	$C_{16}H_{14}O_5$	285.0769	9.12	119.0502	$C_8H_7O^-$	165.0193	$C_8H_5O_4^-$	93.0346	$C_6H_5O^-$
38	Salicylic acid	$C_7H_6O_3$	138.0322	3.97	93.0346	C_6H_5O^1-	65.0397	C_5H_5^1-		
39	Sinapic acid	$C_{11}H_{12}O_5$	224.069	3.00	193.0142	$C_9H_5O_5$^1-	149.0244	$C_8H_5O_3$^1-	121.0295	$C_7H_5O_2$^1-
40	Syringaldehyde	$C_9H_{10}O_4$	182.0585	4.44	151.0037	$C_7H_3O_4$^1-	123.0088	$C_6H_3O_3$^1-	166.0272	$C_8H_6O_4$^1-
41	Syringic acid	$C_9H_{10}O_5$	198.0534	1.58	123.0088	$C_6H_3O_3$^1-	166.9986	$C_7H_3O_5$^1-	182.0221	$C_8H_6O_5$^1-
42	Taxifolin (Dihydro quercetin)	$C_{15}H_{12}O_7$	303.051	5.02	125.0244	$C_6H_5O_3^-$	285.0404	$C_{15}H_9O_6^-$	153.0193	$C_7H_5O_4^-$
43	Tyrosol	$C_8H_{10}O_2$	138.0686	4.38	119.0502	C_8H_7O^1-	93.0346	C_6H_5O^1-	107.0502	C_7H_7O^1-
44	Vanillic acid	$C_8H_8O_4$	168.0428	1.50	108.0217	$C_6H_4O_2$^1-	152.0115	$C_7H_4O_4$^1-	123.0452	$C_7H_7O_2$^1-
45	Vanillin	$C_8H_8O_3$	152.0479	4.54	136.0166	$C_7H_4O_3$^1-	108.0217	$C_6H_4O_2$^1-	92.0268	C_6H_4O^1-

Table S18. R^2 values of calibration curves for all quantified analytes.

A/A	Compound	R^2
1	3-hydroxybenzoic acid	0.9945
2	4-hydroxybenzaldehyde	0.9832
3	4-hydroxybenzoic acid	0.9987
4	Apigenin	0,9885
5	Benzoic acid	0.9987
6	Caffeic acid	0.9990
7	Catechin	0.9970
8	Catechol	0.9976
9	Chlorogenic acid	0.9991
10	Chrysin	0.9883
11	Cinnamic acid	0.9998
12	Diosmetin	0,9987
13	Epicatechin	0.9930
14	Eriodictyol	0.9896
15	Ethyl caffeate	0.9812
16	Ethyl gallate	0.9977
17	Ferulic acid	0.9993
18	Galangin	0,9941
19	Gallic acid	0.9882
20	Gentisic acid	0.9995
21	Hesperetin	0.9893
22	Homovanillic acid	0.9876
23	Hydroxytyrosol	0.9876
24	Kaempferol	0.9819
25	Luteolin	0.9975
26	Myricetin	0.9891
27	Naringenin	0.9978
28	p-coumaric acid	0.9814
29	Pinobanksin	0,9991
30	Pinocembrin	0.9811
31	Protocatechuic acid	0.9824
32	Protocatechuic acid ethyl ester	0.9860
33	Quercetin	0.9938
34	Resveratrol	0.9919
35	Rosmarinic acid	0,9917
36	Rutin	0.9845
37	Sakuranetin	0.9958
38	Salicylic acid	0.9888
39	Sinapic acid	0.9938
40	Syringaldehyde	0.9832
41	Syringic acid	0.9832
42	Taxifolin (dihydroquercetin)	0.9970
43	Tyrosol	0.9938
44	Vanillic acid	0.9902
45	Vanillin	0.9894
